# Microstructure and Transport Properties of CaCl_2_–CaI_2_ Molten Salt: A First-Principles Molecular Dynamics Study

**DOI:** 10.3390/ma19101988

**Published:** 2026-05-11

**Authors:** Muwen Chen, Liguo Zhu, Dengjie Yan, Lingxin Kong, Bin Yang

**Affiliations:** 1 Key Laboratory for Nonferrous Vacuum Metallurgy of Yunnan Province, Kunming University of Science and Technology, Kunming 650093, China; 17773422731@163.com (M.C.); sweams@126.com (D.Y.); 2 State Key Laboratory of Complex Non-Ferrous Metal Resources Clean Utilization, Kunming University of Science and Technology, Kunming 650093, China; 3 The National Engineering Research Center of Vacuum Metallurgy, Kunming University of Science and Technology, Kunming 650093, China; 4 Faculty of Metallurgical and Energy Engineering, Kunming University of Science and Technology, Kunming 650093, China

**Keywords:** first-principles molecular dynamics (FPMD), CaI_2_–CaCl_2_ molten salt, microstructure, transport properties

## Abstract

In this study, first-principles molecular dynamics (FPMD) simulations were employed to systematically investigate the effects of temperature and composition on the microstructure and transport properties of CaCl_2_–CaI_2_ mixed molten salts at the atomic scale. Structural analysis shows that the system exhibits good relaxation behavior and thermodynamic stability, with coordination strength following Ca-Cl > Ca-I. The transport properties reveal a coupled dependence on temperature and composition: increasing CaI_2_ content enhances the diffusion of I^−^ ions, whereas at 1173 K, a decrease in diffusion coefficients is observed for all ionic species. Arrhenius analysis indicates that increasing CaI_2_ content lowers the activation energy for ion migration. The shear viscosity follows the order η(Ca^2+^) > η(Cl^−^) ≥ η(I^−^), and decreases with increasing temperature and CaI_2_ concentration, indicating improved fluidity. Notably, the results reveal a competitive coordination mechanism between Cl^−^ and I^−^ around Ca^2+^, as well as a non-monotonic transport behavior at high temperatures, reflecting the complex coupling between composition and ionic dynamics in mixed halide melts. This study provides a theoretical basis for the optimization of molten salt electrolysis processes and nuclear energy materials, and offers insight for future multiscale simulations and experimental validation.

## 1. Introduction

Molten halides exhibit excellent thermophysical properties and have significant application potential in high-temperature solar power generation [1,2], fuel cells [3], and electrolysis technology [4]. They are among the most promising candidate materials for fourth-generation nuclear reactor designs [5]. The heat storage densities of molten halides are significantly higher than those of nitrates. For example, the NaCl–KCl–CaCl_2_ system has a melting enthalpy of up to 251.37 J/g, and its specific heat capacity remains stable at 0.9–1.1 J/(cm^3^·K) at 1000 K, providing a key material basis for enhancing the energy conversion efficiency of solar systems [6,7]. Molten salts such as CaCl_2_ and CaI_2_ also possess high thermal stability, a wide liquid-phase range, and good ionic conductivity, offering broad application prospects in high-temperature heat transfer, heat storage, and advanced energy systems.

Physical and chemical properties, such as specific heat capacity, thermal conductivity, viscosity, and the electrochemical window, are key parameters for evaluating the applicability of molten salts in high-temperature systems, such as nuclear reactors and solar thermal power generation. These macroscopic properties are determined by the microstructure of the molten salt. Therefore, investigating the intrinsic relationships between the structures of molten halides and their physical and chemical properties is of great theoretical and practical significance in material design and engineering applications.

Owing to the high temperature, toxicity, and strong corrosion of molten salt systems, experimental research faces challenges such as high costs, complex operation, and high safety risks. Molecular dynamics simulations have become an important complementary method for studying the thermophysical properties of molten salts. This approach can reveal the microstructure of molten salts at the atomic scale and accurately predict their macroscopic physical and chemical properties [6,8,9,10,11,12].

Molecular dynamics simulations of molten salts were proposed in 1971. Woodcock and Singer used the Monte Carlo method and Born–Mayer–Huggins and Tosi/Fumi potentials to simulate KCl molten salt [13], and the results were in good agreement with the experimental data. Various rigid and polarizable models have subsequently been used to study the structure and transport properties of alkali halides [14]. In 1983, Tang and Toennies proposed a general dispersion damping function for the Born–Mayer potential, representing the first attempt at describing many-body interactions in multivariate systems [15]. In 1985, Madden and Fowler proposed an asymptotic polarization model, and the LiF simulation results were in good agreement with Hartree–Fock calculation results [16]. In the same year, Car and Parrinello proposed Car–Parrinello molecular dynamics (CPMD), which combines molecular dynamics with density functional theory (DFT) and lays the foundation for simulation research in this field [17]. In 1993, Barnett and Landman implemented DFT-based Born–Oppenheimer molecular dynamics (BOMD), which improved sampling efficiency [18]. Kresse and Hafner proposed a method to initialize CPMD based on energy minimization, which further improved computational efficiency [19]. In 1998, Alfe successfully obtained the diffusion coefficient of liquid aluminum via CPMD, which verified the applicability of DFT in the study of condensed-matter transport properties [20]. In 2003, Aguado et al. parameterized the aspherical ion model through DFT calculations on a multiphase MgO system, which promoted the development of a polarizable model [21]. In 2005, Hazebroucq used tight-binding DFT to study the diffusion behavior of NaCl and KCl [22]. In 2006, Madden et al. noted that dispersion interactions have a major effect on phase-transition behavior [23]. Additionally, Grimme proposed an empirical dispersion correction method for DFT that significantly improved the calculation accuracy [24].

In the field of molten salts, first-principles molecular dynamics (FPMD) is a mainstream simulation method based on BOMD. Compared with traditional molecular dynamics methods, which rely on empirical potential functions and are limited by high parameter dependence and limited transferability, FPMD requires no predefined empirical potentials and offers higher predictive accuracy and portability [25]. FPMD integrates quantum-mechanical calculations (such as DFT) with classical molecular dynamics, enabling simulations to directly describe interatomic interactions at the electronic structure level.

Simulation studies of FPMD in molten salt systems were proposed in 2006. Klix employed CPMD to investigate the diffusion behavior of tritium in FLiBe, marking the first application of ab initio molecular dynamics to molten salt systems and advancing the study of molten salt dynamics into the first-principles era [26]. In 2014, Corradini employed a combined DFT–MD approach to investigate the dispersion effects in LiF, highlighting that dispersion corrections significantly influence the simulated melting points and equilibrium densities [27]. In 2015, Anderson et al. utilized FPMD to simulate FLiNaK and FLiBe molten salts, obtaining thermophysical parameters such as density, diffusion coefficient, and thermal expansion coefficient, which showed good agreement with the experimental data [28].

Over the past decade, research on FPMD of mixed-halide molten salts has increased. In 2019, He et al. employed FPMD simulations to reveal complex ionic-cluster structures within the KF–NaF–AlF_3_ molten salt system and subsequently validated these structures using computational Raman spectroscopy [29]. In 2020, Li et al. employed FPMD simulation techniques to investigate the dynamic interactions between water impurities and molten salts in a MgCl_2_–NaCl–KCl molten salt system, revealing dynamic properties such as energy fluctuations, radial-distribution functions (RDFs), and ion auto-diffusion coefficients [30]. In 2025, Luo et al. investigated the thermophysical properties and microstructures of molten NaCl–KCl–CaCl_2_ salts. Their FPMD simulation results showed good agreement with the experimental data across a temperature range of 873–1173 K, covering parameters such as specific heat capacity, density, self-diffusion coefficient, and viscosity [31].

In this study, FPMD simulations were employed to systematically investigate the synergistic effects of temperature and composition on the microstructure and transport properties of CaCl_2_–CaI_2_ mixed molten salts. The coordination behavior between Ca^2+^ and anions was elucidated by calculating the local structural parameters, including RDFs, coordination number, and bond angle distribution. The transport properties of Ca^2+^, Cl^−^, and I^−^ ions were characterized via kinetic indicators such as the self-diffusion coefficient, activation energy, and shear viscosity. This work provides theoretical support for the optimized design of molten salt electrolysis processes and nuclear energy materials, and lays a foundation for multiscale simulations and experimental verification in related fields.

## 2. Computational Method

In this study, all FPMD simulations were performed using the Vienna ab initio simulation package (VASP, version 5.4.4) [32,33] on a high-performance computing (HPC) cluster. VASP is a commercial software requiring an institutional license. Initial structural models were constructed using Materials Studio 2017 (commercial software) [34], and post-processing analysis, including radial distribution functions (RDF) and mean square displacement (MSD), was carried out using the open-source package VASPKIT (version 1.5.1) [35]. The interaction between ions and electrons was described by the projected augmented wave (PAW) method [36,37], and the electron exchange-correlation energy was treated with the generalized gradient approximation via the Perdew–Burke–Ernzerhof (PBE) functional [38,39]. The valence electron configurations were set to Ca-4s^2^, I-5s^2^5p^5^ and Cl-3s^2^3p^5^. Periodic boundary conditions were applied throughout the simulation to eliminate boundary effects. The lattice parameters of the simulation cell were adjusted based on the experimentally measured density [40], and the initial structure of the molten CaCl_2_–CaI_2_ system was generated using Materials Studio. Each simulation cell contained 180 atoms, with initial positions and velocities randomly assigned according to the specified molar ratios. Two representative CaI_2_ concentrations (20 and 30 mol%) were selected to represent low and intermediate composition regimes. This choice enables a systematic analysis of the competition between Ca-Cl and Ca-I coordination while maintaining the computational feasibility of FPMD simulations. These compositions are also representative of practically relevant concentration ranges in molten halide systems.

The simulations were performed in the NVT ensemble using a Nosé-Hoover thermo-stat [41,42]. Five temperatures (973, 1023, 1073, 1123, and 1173 K) above the melting point were investigated. The van der Waals interactions were corrected using the DFT-D3 method with Becke–Johnson damping [43,44]. The plane-wave cutoff energy was set to 400 eV, a 1 × 1 × 1 k-point grid was used. The integration time step was 1 fs, and the total simulation time per temperature point was 5 ps. It is noted that this simulation time is sufficient to obtain converged structural properties (e.g., RDF and ADF after equilibration), whereas transport properties such as diffusion coefficients and viscosity may still exhibit statistical uncertainty due to finite trajectory length.

Classical molecular dynamics simulations were not performed in this study due to the difficulty in constructing reliable and transferable force field parameters for the CaCl_2_–CaI_2_ system, particularly due to the highly polarizable nature of I^−^ and the presence of competing coordination environments.

## 3. Density Measurements

Density measurement methods include Archimedes, gas expansion, specific-gravity, and X-ray diffraction methods. The CaCl_2_–CaI_2_ molten salt system is highly volatile and corrosive; therefore, this study used the Archimedes method, which is based on the hydrostatic balance principle, to measure the density [45,46]. According to Archimedes’ law, the buoyancy of an object immersed in a liquid is equal to the weight of the displaced liquid, which enables reliable density measurements of molten halides.

In this method, the volume of the tungsten hammer used for the determination can be obtained by measuring its mass in air and pure water using the following formula:

(1) $$V_{0}\;=\;\frac{M\;-{\;M}_{0}}{\rho_{0}}$$

where V_0_ is the volume of the tungsten hammer; M is the mass of the hammer in air; and M_0_ is the mass of the hammer in a liquid with a density of ρ_0_. Thus, the density of the high-temperature melts can be calculated as follows:

(2) $$\rho \;=\;\frac{M\;-{\;M}_{1}}{V}$$

where ρ is the density of the high-temperature melt; V is the corrected volume of the tungsten hammer; and M_1_ is the mass of the hammer in the high-temperature melt. The mass was weighed using a ten-thousandth precision balance (using a hook at the bottom of the balance to hang the tungsten wire connected to the tungsten hammer).

The volume of the tungsten hammer measured at room temperature changes at high temperatures; therefore, a thermal expansion correction must be applied. Considering the linear thermal expansion coefficient of tungsten, α = 4.6 × 10^−6^ K^−1^ [47], the corrected volume at the measurement temperature is corrected as follows:

(3) $$V\;={\;V}_{0}\;+\;3\alpha {\cdot V}_{0}\left(T_{2}\;-\;T_{1}\right),$$

where V_0_ is the volume calibrated with pure water at temperature T_1_, and T_2_ is the temperature of the molten salt during the density measurement.

Pretreated CaI_2_ and CaCl_2_ salts (purity ≥99%, Wuhan Huaxiang, China) were dehydrated in a vacuum furnace at 473 K for 24 h. The dried salts were thoroughly mixed in an inert-gas atmosphere based on the target molar ratios and transferred to an alumina crucible inside the reactor. After sealing, the reactor was evacuated to 5–10 Pa and heated to 1073 K at a rate of 10 K·min^−1^ in an argon atmosphere. The density measurements commenced after the temperature was stabilized for 30 min.

**Figure 1:**
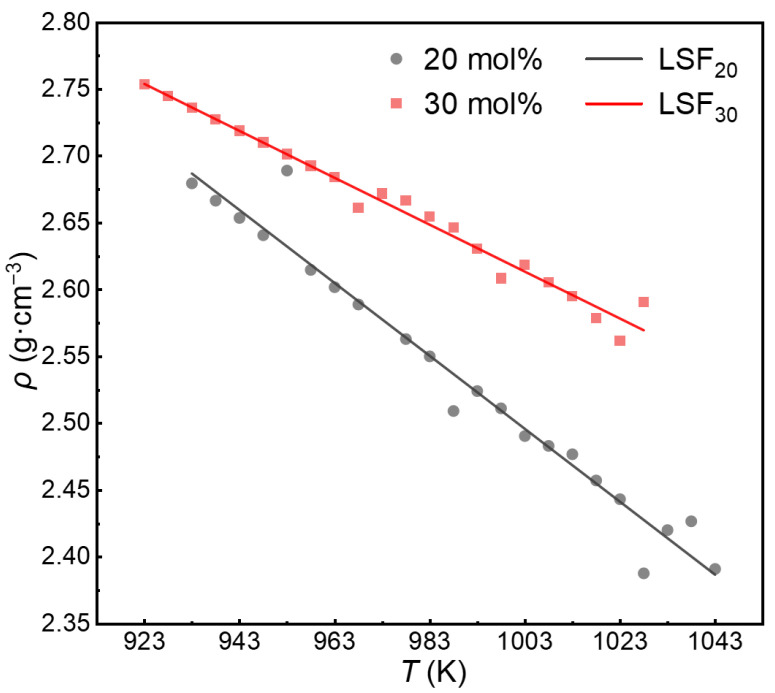
Density of the CaCl_2_–CaI_2_ molten salt system at different CaI_2_ contents.

A CaCl_2_–CaI_2_ molten salt system with a total mass of 500 g was used as the sample. Density variations in the systems with CaCl_2_:CaI_2_ molar ratios of 7:3 and 8:2 were investigated at 923–1043 K. The results are shown in Figure 1. As shown in Figure 1, for a given composition, the density decreased with increasing temperature and was linearly correlated with the temperature. Least squares fitting (LSF) was used to obtain the fitting line as follows:

(4) $${\mathrm{LSF}}_{20}\;=\;3.9825\;-\;0.00176T\;\;\;\;R^{2}\;=\;0.97988,$$

(5) $${\mathrm{LSF}}_{30}=4.6239-0.00273T\;\;\;\;R^{2}=0.96563$$

In this context, LSF_20_ and LSF_30_ denote the fitted density values of the molten salts with CaI_2_ contents of 20 and 30 mol%, respectively. At a given temperature, the density increased with an increasing CaI_2_ mole fraction. A coefficient of determination (R^2^) close to 1 indicates an excellent linear fit of the experimental density–temperature data, and statistical analysis confirms that the slope is highly significant (*p* < 0.001). The accurate density measurements provide an experimental basis for establishing the correspondence between the number of atoms and simulation cell volume in the system, and support the parameterization and validation of subsequent FPMD simulations.

## 4. Results and Discussion

### 4.1. Microstructure

**Figure 2:**
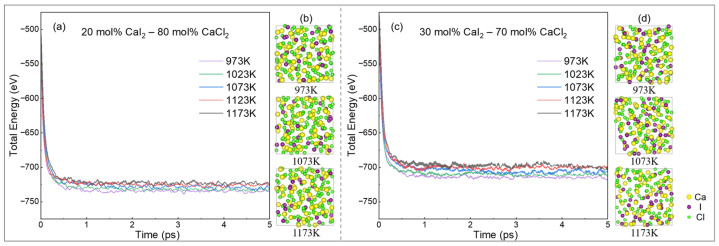
Dynamic stability of the CaCl_2_–CaI_2_ molten salt system: (**a**,**b**) total energy evolution and corresponding atomic configuration snapshots of the 20 mol% CaI_2_ system; (**c**,**d**) total energy evolution and corresponding atomic configuration snapshots of the 30 mol% CaI_2_ system.

The structural stability evaluation of the molten salt system is a prerequisite for kinetic analysis. Figure 2 shows the total energy evolution and atomic structure characteristics of the CaCl_2_–CaI_2_ molten salt system under two composition ratios and different temperature conditions. Figure 2a,c show that the total energy of the system converged rapidly at the beginning of the simulation under all temperature conditions and maintained small fluctuations after reaching equilibrium. The absence of energy divergence or structural instability indicates that a simulation time of 5 ps is sufficient for the molten salt system to reach equilibrium. As the temperature increased, the total equilibrium energy of the system gradually decreased (i.e., its negative value increased), which was consistent with the trend of the energy-state variation at high temperatures. Additionally, an increase in calcium-iodide content increased the total equilibrium energy of the system.

Figure 2b,d show snapshots of the atomic configurations at the corresponding temperatures. The ions maintain a disordered liquid distribution after reaching equilibrium, with no unstable behavior such as local crystallization or phase separation. Combined with energy evolution and structural analysis, the system rapidly converged (stabilized within approximately 1–2 ps) under different composition ratios and temperature conditions, indicating the excellent structural relaxation capability and thermodynamic stability of the molten salt system.

The experimentally measured densities (Figure 1) were used to determine the lattice parameters and initial cell volumes of the FPMD simulations at each temperature and composition, ensuring that the simulated systems reproduce the correct macroscopic density. In this way, the atomistic models are directly constrained by experimental thermodynamic data, providing a physically consistent starting point for structural and dynamical analyses.

Furthermore, the experimental density shows a linear decrease with increasing temperature, reflecting thermal expansion of the molten CaCl_2_–CaI_2_ system. This macroscopic behavior is consistent with the microscopic structural evolution observed in the present simulations. As will be shown in the following RDF analysis, increasing temperature leads to a broadening of the first coordination shell and a shift in peak positions toward larger interatomic distances, indicating a weakening of short-range ordering.

Therefore, the experimental density not only provides input constraints for the simulation setup but also offers an indirect validation of the structural response obtained from the atomistic model.

**Figure 3:**
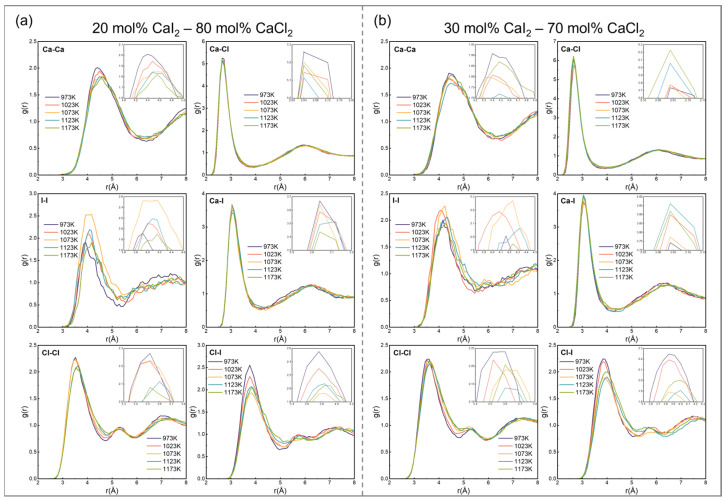
The radial distribution functions of ion pairs in CaCl_2_–CaI_2_ molten salt at different CaI_2_ contents and temperatures are: (**a**) 20 mol% CaI_2_, (**b**) 30 mol% CaI_2_ at 973, 1023, 1073, 1123 and 1173 K.

The RDFs characterize the spatial distribution of specific particle pairs (i and j) in a multi-component system. The value of the function *g*_ij_(*r*) at distance *r* reflects the local distribution probability of particle j relative to the reference particle i at that distance. This was calculated by statistically averaging molecular dynamics simulation trajectories, as shown in Equation (6) [48]:

(6) $$g_{\mathrm{ij}}\left(r\right)\;=\;\frac{1}{4\pi {r^{2}\rho }_{j}N_{i}}\left[\frac{\langle n_{\mathrm{ij}}\left(r\right)\rangle }{\Delta r}\right]$$

where *N*_i_ denotes the total number of reference particles i; *ρ*_j_ represents the particle number density of particle j in the system [49]; Δ*r* is the interval width derived from the maximum cutoff distance *r*_max_; and 〈*n*_ij_(*r*)〉 is the average number of i − j particle pairs observed within the Δ*r* interval. The RDFs results for the CaCl_2_–CaI_2_ molten salt system obtained from the FPMD simulations after 5 ps are shown in Figure 3.

Figure 3 shows that when the temperature increased above the melting point, the RDFs curves exhibited typical molten salt structural characteristics: oscillatory decay, highest intensity at the first coordination peak, gradual weakening as the radial distance increased, and convergence to unity, indicating short-range order and long-range disorder in the system. The RDFs peaks of anion–cation pairs (e.g., Ca-Cl and Ca-I) and like-charge ion pairs (e.g., Cl-Cl and I-I) appear alternately, suggesting a layered arrangement of cations and anions around a central ion, a structural feature dominated by Coulomb interactions. The first peak intensity of oppositely charged ion pairs (e.g., Ca-Cl) was much greater than that of like-charged pairs (e.g., Cl-Cl), reflecting stronger electrostatic attraction and a tighter coordination structure [50]. Moreover, the first peak of the anion–cation pairs occurred at a smaller radial distance, suggesting that Coulomb attraction leads oppositely charged ions to preferentially occupy the nearest-neighbor coordination shell [51]. The RDFs analysis of different ion pairs suggests that the coordination environment of similar ion types remains relatively stable. The first peak position decreased in the order of Ca-Ca > I-I > Cl-I > Cl-Cl > Ca-I > Ca-Cl, whereas the first peak intensity decreased in the order of Ca-Cl > Ca-I > I-I ≈ Cl-I > Cl-Cl > Ca-Ca. The strength, position, and steepness of the first peak indicate that the electrostatic attraction between anions and Ca^2+^ follows the order Cl^−^ > I^−^, which is attributed to the smaller ionic radius of Cl^−^ and its stronger Coulomb interaction with cations.

As the temperature increased from 973 to 1173 K, the first peak position of the RDFs shifted to a greater distance and the peak intensity decreased, indicating that the increase in thermal disturbance weakened the short-range order of the molten salt. This phenomenon was consistent across all ion pairs, reflecting the general perturbation effect of increasing temperature on the local structure.

When the temperature was constant, increasing the CaI_2_ content did not cause noticeable shifts in the first peak positions of the Ca-Cl and Ca-I RDFs curves, indicating that the equilibrium bond lengths of these anion–cation pairs were not affected by composition changes. Although the intensity of the first Ca-I peak increased slightly, its position remained stable. In contrast, the RDFs of the Cl-I and I-I ion pairs showed clear dependence on the composition. With increasing CaI_2_ content, the first peak shifted to greater radial distances, the peak intensity decreased progressively, and the structural order was reduced, further confirming the greater stability of the Ca-Cl bonds. Furthermore, the shoulder peaks in the Cl-Cl and Cl-I RDFs curves suggest the presence of an additional short-range ordered coordination configuration in the system, which may be owing to the formation of stable complex ion clusters such as CaCl_6_^4−^ by Cl^−^ ions.

To explore the specific form of the anion–cation structure, this study systematically analyzed the coordination behavior between ions via coordination number (CN) curves, the potential of the mean force, and the bond angle distributions. The CN is defined as the average number of particles j within a sphere of radius r_min_ centered on the reference particle i, where r_min_ corresponds to the position of the first minimum in the RDFs, as shown in Equation (7):

(7) $$N_{\mathrm{ij}}\left(r\right)\;=\;4\pi \rho_{j}\int_{0}^{r_{\min}}g_{\mathrm{ij}}\left(r\right)r^{2}dr$$

where r_min_ is the position of the first minimum in the RDFs, which corresponds to the region of minimum probability between the first and second coordination shells (first and second peaks, respectively). This minimum serves as a boundary to distinguish nearest-neighbor and next-nearest-neighbor atoms.

**Figure 4:**
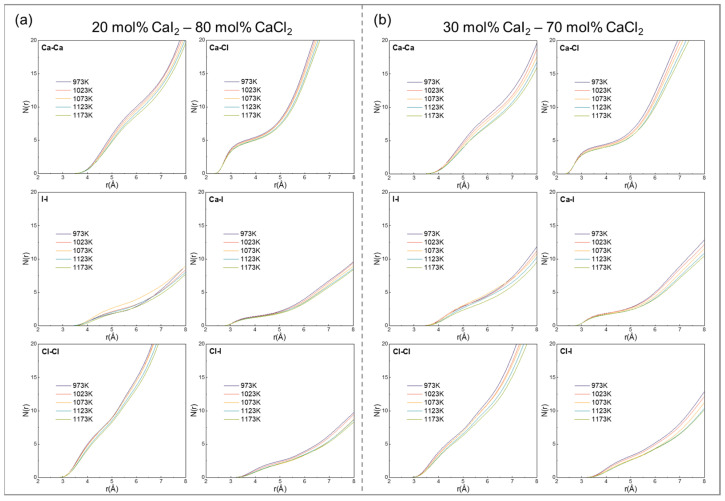
The coordination number of ion pairs in CaCl_2_–CaI_2_ molten salt at different CaI_2_ contents and temperatures: (**a**) 20 mol% CaI_2_, (**b**) 30 mol% CaI_2_ at 973, 1023, 1073, 1123 and 1173 K.

Figure 4 shows the coordination number (CN) profiles of different ion pairs in the CaCl_2_–CaI_2_ molten salt system. The CN curves for cation–anion pairs exhibit plateau-like features, where higher plateau values generally indicate stronger local ion association and more persistent coordination environments [52].

The relative magnitudes of the CN plateaus suggest that Ca^2+^ tends to exhibit a stronger coordination with Cl^−^ compared to I^−^ under the present conditions. This difference can be qualitatively related to the smaller ionic radius of Cl^−^ and its stronger electrostatic interaction with Ca^2+^.

With increasing temperature, the CN curves shift downward and the plateau values gradually decrease. For example, at 30 mol% CaI_2_, the plateau value for the Ca-Cl pair decreases from approximately 4.5 at 973 K to 4.0 at 1173 K, corresponding to a reduction of ~11.1%. This indicates that thermal fluctuations progressively weaken the persistence of local coordination environments and broaden the distribution of interatomic distances, leading to less well-defined coordination shells.

At a fixed temperature, an increasing CaI_2_ content results in modest variations in the CN plateau values. In particular, Ca-I coordination shows a slight increase, while Cl-I and I-I correlations exhibit a decrease. These trends suggest a redistribution of local coordination environments associated with changes in anion composition, reflecting a competition between different anion species in coordinating Ca^2+^ and thereby modifying the short-range structural organization.

To verify the interaction mechanism between anions and cations, the RDFs was used to calculate the potential of the mean force (PMF) for the ion pairs. The PMF is calculated as follows [53,54]:

(8) $${W_{\mathrm{ij}}}^{\mathrm{eff}}\left(r\right)\;=\;-k_{B}T\ln g_{\mathrm{ij}}\left(r\right)$$

where *W*_ij_^eff^(*r*) represents the effective interaction potential between particles i and j (unit: kcal/mol), k_B_ is the Boltzmann constant (unit: 1.3806505 × 10^−23^ J·K^−1^), *T* is the system temperature (unit: K), and *g*_ij_(*r*) is RDFs of the ion pair. In the PMF curve, the energy barrier between the first minimum and maximum reflects the energy required for an ion to escape from the first coordination shell. A higher barrier indicates a greater ion-pair binding stability.

**Figure 5:**
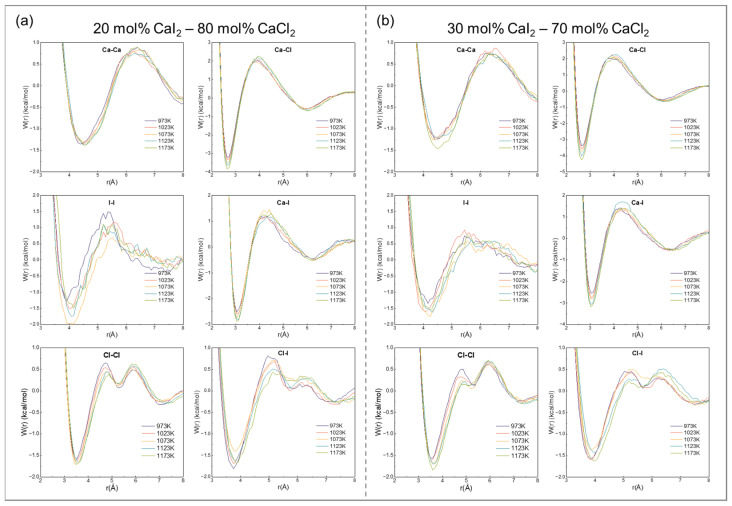
The average force potential of ion pairs in CaCl_2_–CaI_2_ molten salt at different CaI_2_ contents and temperatures: (**a**) 20 mol% CaI_2_, (**b**) 30 mol% CaI_2_ at 973, 1023, 1073, 1123 and 1173 K.

Figure 5 shows the PMF curves of different ion pairs in the CaCl_2_–CaI_2_ molten salt system. The energy barrier of the PMF decreased in the order Ca-Cl > Ca-I > Ca-Ca, indicating that the interaction between Cl^−^ and Ca^2+^ was the strongest, followed by I^−^, whereas the interaction between the Ca^2+^-ion pairs was the weakest. This order was consistent with the smaller ionic radius of Cl^−^ and stronger Coulomb attraction it induces.

As the temperature increased, the PMF energy barriers for the Ca-Cl and Ca-I pairs showed little change, indicating that thermal disturbance had a limited direct effect on the binding strength. This result further confirmed that the stability of the ion-pair interactions is governed primarily by the local coordination structure.

When the temperature was constant, increasing the CaI_2_ content increased the PMF energy barrier for the Ca-Cl and Ca-I pairs, indicating a weakening of the effective interionic force. This weakening occurred because a higher I^−^ ratio intensified local coordination competition, thereby reducing the binding strength of the original ion pair.

The bond angle distribution function revealed the characteristics and evolution of the anion–cation coordination configurations microscopically. This provided an effective method for determining the coordination configurations of ions. This calculation is as follows:

(9) $$\theta_{\mathrm{jik}}\;=\cos^{-1}\frac{r_{\mathrm{ij}}^{2}+r_{\mathrm{ik}}^{2}-r_{\mathrm{jk}}^{2}}{2r_{\mathrm{ij}}r_{\mathrm{ik}}}$$

where i denotes a Ca^2+^ ion, and j and k are distinct Cl^−^ or I^−^ ions within a distance r_min_ of the Ca^2+^ ion.

**Figure 6:**
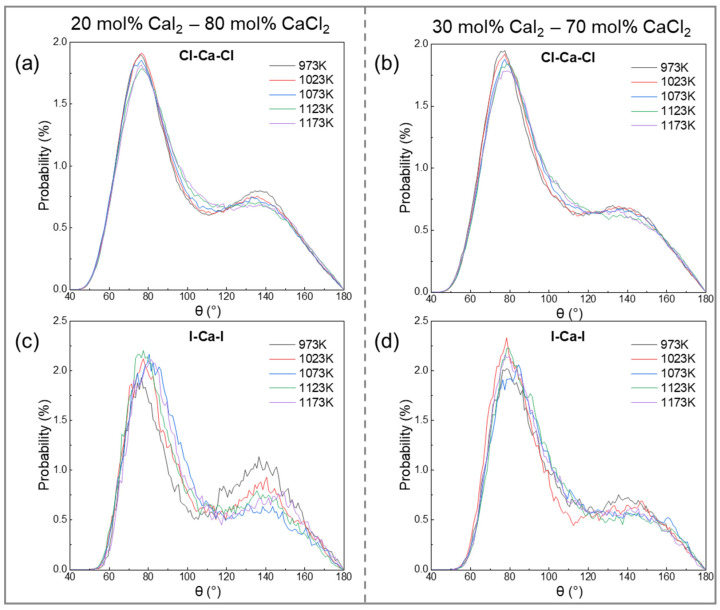
The bond angle distribution functions of Cl-Ca-Cl and I-Ca-I in CaCl_2_–CaI_2_ molten salt at different CaI_2_ contents and temperatures: (**a**) 20 mol% CaI_2_ of Cl-Ca-Cl, (**b**) 30 mol% CaI_2_ of Cl-Ca-Cl, (**c**) 20 mol% CaI_2_ of I-Ca-I, (**d**) 30 mol% CaI_2_ of I-Ca-I.

Figure 6 shows the bond angle distributions of Cl-Ca-Cl and I-Ca-I in the CaCl_2_–CaI_2_ molten salt system. The principal peaks of both distributions lie in the range of 60–100°, with a maximum near 80°, which is slightly lower than the ideal tetrahedral or octahedral coordination angle of 90° [55]. This indicates that the coordination configuration between Ca^2+^ and the anions deviates from the ideal geometry, resulting in the adoption of a distorted local structure.

As shown in Figure 6a,b, the Cl-Ca-Cl distribution exhibits a broad curve and low peak, indicating significant configurational fluctuations and low local symmetry. As the temperature increased from 973 K to 1173 K, the peak height decreased, and the distribution broadened further, reflecting enhanced bond angle fluctuations due to thermal disturbance. Increasing the CaI_2_ content has little effect on the peak position of the Cl-Ca-Cl distribution (offset <2°), demonstrating that the Cl^−^ coordination mode has strong structural stability and environmental adaptability.

As shown in Figure 6c,d, although the main peak of the I-Ca-I distribution appeared at a similar angle, it was higher and sharper than that of the Cl-Ca-Cl distribution, indicating a more ordered coordination configuration between I^−^ and Ca^2+^. This feature may be attributed to the steric hindrance caused by the larger ionic radius of I^−^, where its diffuse electron cloud constrained the bond angles into a narrower distribution. The broader broadening and greater peak reduction in the I-Ca-I distribution, compared with Cl-Ca-Cl, point to a higher sensitivity of the I^−^ coordination to thermal disturbance. Increasing the CaI_2_ content increased the peak intensity and narrowed the distribution, indicating enhanced I^−^ coordination competition and improved local structural order.

The comprehensive analysis shows that although both Cl^−^ and I^−^ tend to form an 80° bond angle with Ca^2+^, the I^−^ coordination configuration results in a higher order and greater temperature sensitivity, whereas Cl^−^ coordination results in greater structural stability.

### 4.2. Transport Properties

The mean square displacement (MSD) is a key parameter for characterizing the atomic diffusion behavior in molten salt systems. It is defined as the mean square of atomic displacement over a given time interval. The calculation is given by Equation (10):

(10) $$\mathrm{MSD}\;={\;\left[∆r\left(t\right)\right]}^{2}\;=\;\frac{1}{N}\sum_{t}{\left|r_{i}\left(t\right)-r_{i}\left(0\right)\right|}^{2}$$

where *r*_i_(*t*) is the position of particle i at time *t* and N is the total number of atoms in the system. When the temperature exceeds the melting point, the MSD varies linearly with time, following Einstein’s diffusion law [56], and its slope directly reflects the atomic diffusion coefficient.

**Figure 7:**
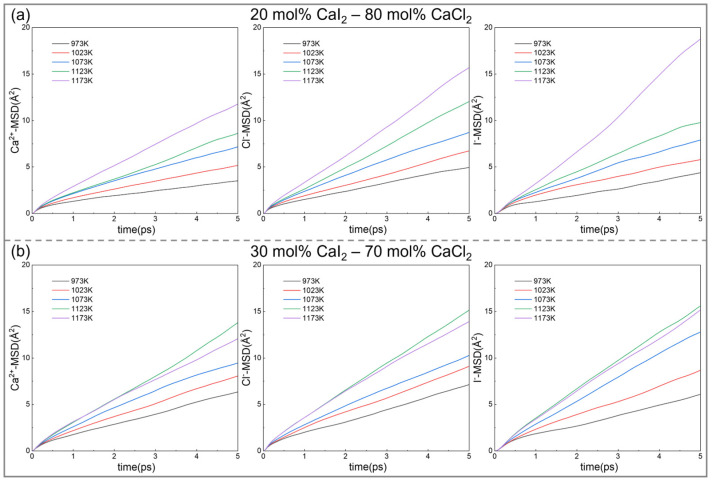
The MSD of ions in CaCl_2_–CaI_2_ molten salt at different CaI_2_ contents and temperatures: (**a**) 20 mol% CaI_2_, (**b**) 30 mol% CaI_2_ at 973, 1023, 1073, 1123 and 1173 K.

Figure 7 shows the time-dependent MSDs of Ca^2+^, Cl^−^, and I^−^ ions in the CaCl_2_–CaI_2_ molten salt system. Under all temperature and composition conditions, the MSD increased linearly with time, which is consistent with the diffusion behavior of high-temperature melts. As the temperature increased from 973 to 1173 K, the slopes of the MSD curves for all ions increased, reflecting an enhanced atomic thermal motion and ion-migration ability.

When the temperature was constant, increasing the CaI_2_ content increased the MSD slopes of the Ca^2+^, Cl^−^, and I^−^ ions at 973–1123 K, indicating that a higher CaI_2_ ratio promoted ion diffusion. However, at 1173 K, the MSD slopes of Ca^2+^ and Cl^−^ remained almost unchanged, whereas that of I^−^ decreased, suggesting that the diffusion of I^−^ was suppressed at high temperatures. This may be related to local structural rearrangements and changes in interionic interactions.

MSD analysis demonstrated that the temperature and composition significantly affected ion diffusion in the CaCl_2_–CaI_2_ molten salt system. Increasing the temperature generally enhanced ion diffusion, whereas increasing the CaI_2_ content promoted diffusion between 973 K and 1123 K, but suppressed I^−^ diffusion at 1173 K.

The self-diffusion coefficient *D* can be expressed as follows:

(11) $$D\;=\;\underset{t\to \infty }{\lim}\frac{1}{6}\frac{{d\left[∆r\left(t\right)\right]}^{2}}{dt}$$

where a factor of 1/6 was introduced to account for the equivalence of the six diffusion directions. Thus, the self-diffusion coefficient of a particle was calculated to one-sixth of the slope of the linear region of the MSD curve. Table 1 presents the fitted slope values and coefficients of determination (R^2^) for the MSD in the linear region (1–5 ps) at different temperatures for each component.

**Figure 8:**
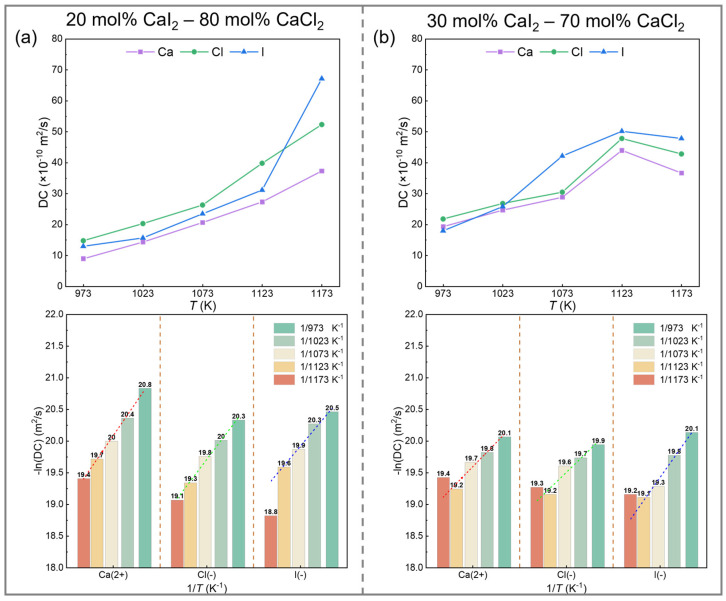
The self-diffusion coefficients of ions in CaCl_2_–CaI_2_ melts at different CaI_2_ contents and temperatures: (**a**) 20 mol% CaI_2_, (**b**) 30 mol% CaI_2_ at 973, 1023, 1073, 1123 and 1173 K.

Figure 8 shows the variation in the self-diffusion coefficients of Ca^2+^, Cl^−^, and I^−^ ions with CaI_2_ content in the CaCl_2_–CaI_2_ molten salt system over a temperature range of 973–1173 K. As shown in Figure 8, at a CaI_2_ concentration of 20 mol%, the self-diffusion coefficients followed the order *D*(Cl^−^) > *D*(I^−^) > *D*(Ca^2+^) at 973–1123 K, shifting to *D*(I^−^) > *D*(Cl^−^) > *D*(Ca^2+^) at 1173 K. At a CaI_2_ concentration of 30 mol%, the order of self-diffusion coefficients is *D*(Cl^−^) > *D*(Ca^2+^) > *D*(I^−^) in the temperature range of below 1023 K. When the temperature increased to 1073 K, the order was *D*(I^−^) > *D*(Cl^−^) > *D*(Ca^2+^). The above results show that the diffusion ability of I^−^ is significantly enhanced with the increase in temperature, and its sensitivity to temperature is higher than that of Cl^−^ and Ca^2+^. Increasing the CaI_2_ content increases the self-diffusion coefficient of all ions in the temperature range of 973–1123 K, but decreases the self-diffusion coefficient at 1173 K.

When the molten salt composition was fixed, the relationship between the ion self-diffusion coefficient and temperature conformed to the Arrhenius equation, as follows:

(12) $$D\;={\;D}_{0}\exp\left(-\frac{E_{a}}{RT}\right)$$

The activation energy Ea and pre-exponential factor D_0_ obtained by fitting are listed in Table 2. The coefficient of determination (R^2^) for all linear fits exceeds 0.9, suggesting that the Arrhenius model reasonably describes the temperature dependence of the diffusion coefficients within the studied range. Although the MSD curves exhibit a clear linear regime corresponding to diffusive behavior, the finite simulation time (5 ps) may introduce statistical uncertainty in the calculated diffusion coefficients. Therefore, the reported values are primarily used to analyze relative trends rather than absolute magnitudes.

Table 2 summarizes the Arrhenius fitting parameters for ion diffusion in CaCl_2_–CaI_2_ molten salts with different compositions. The results indicate that the variation in diffusion activation energy is ion-dependent. Specifically, the activation energies of Ca^2+^ and Cl^−^ decrease with increasing CaI_2_ content, whereas I^−^ exhibits a weaker and non-monotonic variation. This behavior suggests that the diffusion of I^−^ is governed by a combination of thermal activation and local structural heterogeneity arising from its distinct coordination environment. In particular, the increasing competition between Cl^−^ and I^−^ for coordination around Ca^2+^ at higher CaI_2_ concentrations may induce local structural frustration, which modulates ion migration pathways. The overall decrease in activation energy reflects a progressive reorganization of the local coordination structure with composition, which facilitates ion transport. Notably, the self-diffusion coefficients of Ca^2+^, Cl^−^, and I^−^ decrease at 1173 K and 30 mol% CaI_2_, which may be associated with transient cluster formation and dissociation processes at elevated temperatures, indicating a possible modification of diffusion mechanisms under these specific thermodynamic conditions.

**Figure 9:**
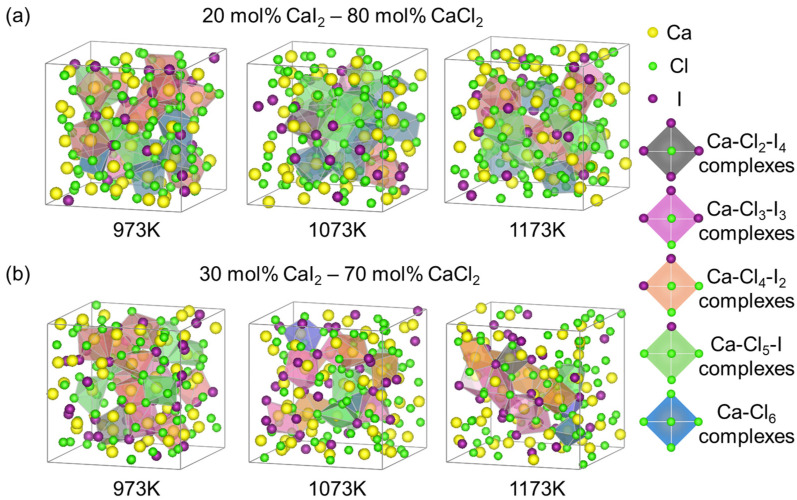
The local ionic structure of CaCl_2_–CaI_2_ molten salt at different CaI_2_ contents and temperatures: (**a**) 20 mol% CaI_2_, (**b**) 30 mol% CaI_2_ at 973, 1073 and 1173 K.

Figure 9 illustrates the evolution of Ca^2+^ coordination environments with temperature in the CaCl_2_–CaI_2_ mixed molten salt system. When the CaI_2_ content was increased to 30 mol% (Figure 9b), the relative population of Ca-Cl coordinated clusters decreased, accompanied by an increase in Ca-I coordination motifs.

At 973 K, Ca-centered polyhedral units are mainly characterized by Ca-Cl-dominated octahedral-like coordination environments, including mixed-halide species such as CaCl_5_I^4−^ and CaCl_4_I_2_^4−^ (illustrated by green and orange polyhedra in Figure 9b). As temperature increases from 973 K to 1173 K, partial substitution of Cl^−^ by I^−^ is observed in the first coordination shell, leading to the formation of more iodine-rich mixed coordination environments such as CaCl_3_I_3_^4−^ and CaCl_2_I_4_^4−^ (purple and gray polyhedra).

At 1173 K, iodine-containing coordination environments become more prevalent, indicating a redistribution of anion species around Ca^2+^ under enhanced thermal motion. This change reflects the increased configurational flexibility of the local coordination structure at elevated temperatures.

In contrast, the 20 mol% CaI_2_ system (Figure 9a) exhibits a more chlorine-dominated coordination environment at 973 K, where CaCl_6_^4−^-like octahedral configurations are more frequently observed (blue polyhedra). With increasing temperature, I^−^ gradually participates in the first coordination shell, leading to a progressive evolution toward mixed-halide coordination motifs.

These results highlight that both temperature and composition play important roles in modulating the local coordination environment of Ca^2+^ ions. The observed structural evolution is expected to influence ion transport behavior through changes in local coordination flexibility and anion distribution, although a detailed quantitative correlation requires further analysis.

From a practical perspective, intermediate CaI_2_ contents and moderate temperatures appear to provide a balanced coordination environment where both structural order and configurational flexibility coexist, which may be favorable for ionic transport in molten salt systems.

**Figure 10:**
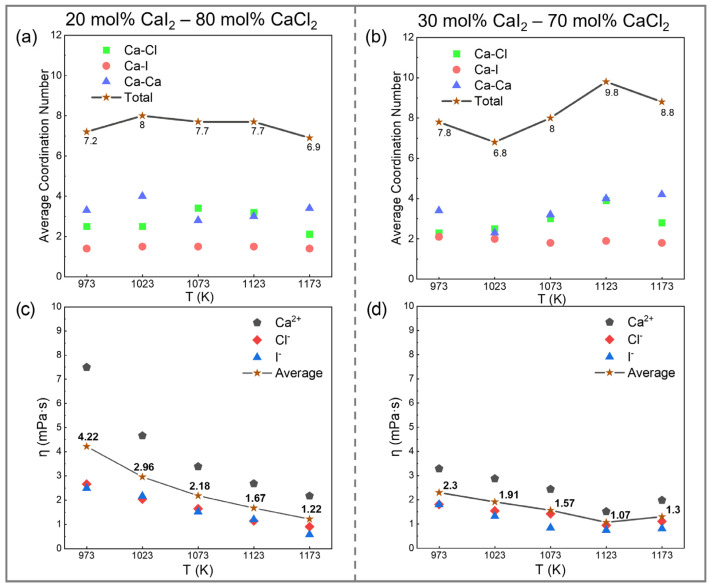
The ion coordination distribution and shear viscosity of CaCl_2_–CaI_2_ molten salt at different CaI_2_ contents and temperatures: (**a**) average CN of Ca^2+^ at 20 mol% CaI_2_ and (**b**) 30 mol% CaI_2_. (**c**) Shear viscosity at 20 mol% CaI_2_ and (**d**) 30 mol% CaI_2_.

In studies of melt properties, the Stokes–Einstein relation describes the functional relationship between the shear viscosity and self-diffusion coefficient, as given in Equation (13):

(13) $$\eta_{i}\;=\;\frac{k_{B}T}{6\pi R_{i}D_{i}}$$

where *η*_i_ is the shear viscosity of ion i (unit: Pa·s), *R*_i_ is the ionic radius (unit: m), *T* is the temperature (unit: K), k_B_ is the Boltzmann constant (unit: 1.3806505 × 10^−23^ N·m·K^−1^), and *D*_i_ is the ion self-diffusion coefficient (unit: m^2^·s^−1^). Table 3 lists the radii of the Ca^2+^, Cl^−^, and I^−^ ions under different coordination states. The ion trajectory and coordination environment were calculated and analyzed, and the results are shown in Figure S1. Based on the average coordination number of Ca^2+^, the corresponding ionic radius was selected. Using Equation (13), the shear viscosity of individual ions and the overall system was estimated as a function of temperature, as shown in Figure 10c,d. It should be noted that the Stokes–Einstein (SE) relation is an approximate hydrodynamic model and may not be strictly valid in strongly interacting ionic systems such as molten salts, where ion–ion correlations and coordination-dependent dynamics can introduce deviations. Therefore, the viscosity values reported here should be regarded as semi-quantitative estimates, mainly useful for trend analysis. In addition, since viscosity is derived from diffusion coefficients obtained from finite simulation trajectories, it is subject to the same statistical uncertainty.

It should be noted that the SE relation is an approximate approach based on hydrodynamic assumptions, which may not be strictly valid in strongly interacting ionic systems such as molten salts. In particular, ion–ion correlations and coordination-dependent dynamics can lead to deviations from ideal SE behavior. Therefore, the viscosity values reported here are intended to provide qualitative to semi-quantitative insights into relative trends, rather than precise absolute values.

Within this framework, the estimated results suggest that the shear viscosity of both compositions decreases with increasing temperature, showing a typical negative temperature dependence. Among different ionic species, the viscosity follows the general trend *η*(Ca^2+^) > *η*(Cl^−^) ≥ *η*(I^−^), which can be attributed to the combined influence of ionic charge and size.

Similarly, in the temperature range of 973–1123 K, the estimated viscosity tends to decrease with increasing CaI_2_ content, indicating enhanced fluidity. At 1173 K, a slight increase in viscosity is observed. This behavior may be related to temperature-induced structural fluctuations and transient coordination rearrangements, although further analysis is required to confirm this mechanism.

Overall, these results suggest that the incorporation of I^−^ generally promotes fluidity in the intermediate temperature range, while its effect becomes less pronounced at higher temperatures. The consistency between structural evolution (e.g., coordination environment and RDF) and transport trends further supports the qualitative validity of this analysis.

### 4.3. Implications for Structural–Transport Coupling

To bridge the microscopic structural evolution with macroscopic transport behavior, the present results suggest that coordination competition between Ca-Cl and Ca-I interactions may influence the properties of the CaCl_2_–CaI_2_ molten salt system. The gradual substitution of Cl^−^ by I^−^ modifies the local coordination environment of Ca^2+^, leading to changes in polyhedral connectivity and local structural flexibility, which are likely related to variations in ion transport behavior at intermediate temperatures.

At higher I^−^ contents and elevated temperatures, the coordination environment becomes more dynamically heterogeneous, which may transiently influence the continuity of diffusion pathways. Although only two compositions were explicitly investigated, the consistent trends observed in coordination number, PMF, diffusion coefficients, and viscosity suggest a smooth and physically continuous dependence on composition.

On this basis, a compositional design window rather than a single optimal point can be inferred. In particular, a CaI_2_ content in the range of approximately 20–30 mol% appears to provide a favorable balance between structural stability and ionic mobility. This indicates that the transport performance of the molten salt is likely governed by a competition between relatively rigid Ca-Cl coordination networks and more flexible Ca-I coordination environments, rather than a simple monotonic dependence on composition.

## 5. Conclusions

In this study, the structural evolution and transport properties of a CaCl_2_–CaI_2_ molten salt system at 973–1173 K were systematically investigated via FPMD simulations. The main conclusions are as follows:

(1) The melt exhibits short-range order and long-range disorder. The coordination strength between Ca^2+^ and the anions follows the order Ca-Cl > Ca-I, with a distorted octahedral coordination environment. Compared with Cl-Ca-Cl, the I-Ca-I angular distribution shows relatively stronger structural ordering but higher sensitivity to temperature. Increasing CaI_2_ content enhances the competition between Ca-Cl and Ca-I coordination, leading to reduced ordering in Cl-I and I-I correlations, while the Ca-Cl interaction remains relatively stable. These results suggest that local coordination structure plays an important role in governing ion dynamics.
(2) Ion diffusion exhibits a coupled dependence on composition and temperature. At 20 mol% CaI_2_, the diffusion coefficients follow *D*(Cl^−^) > *D*(I^−^) > *D*(Ca^2+^) at 973–1123 K, shifting to *D*(I^−^) > *D*(Cl^−^) > *D*(Ca^2+^) at 1173 K. When the CaI_2_ content increases to 30 mol%, I^−^ shows higher mobility than Cl^−^ over a broader temperature range, while at 1173 K, the diffusion of all ions is slightly suppressed. Arrhenius analysis indicates that an increasing CaI_2_ content reduces the activation energy for ion migration. Structural analysis suggests that the presence of I^−^ modifies the local coordination environment around Ca^2+^, which correlates with the observed changes in diffusion behavior.
(3) Based on the Stokes–Einstein relation, the shear viscosity follows *η*(Ca^2+^) > *η*(Cl^−^) ≥ *η*(I^−^). In the temperature range of 973–1123 K, the introduction of I^−^ improves the fluidity of the molten salt. However, at 1173 K, this effect becomes less pronounced.

This study elucidates the coupled influence of temperature and composition on the structure and transport properties of CaCl_2_–CaI_2_ molten salts at the atomic scale, providing insights for the design of molten salt systems in electrochemical and nuclear applications. It should also be noted that, due to the finite simulation time (5 ps), the reported transport properties may contain statistical uncertainties and are therefore mainly discussed in terms of qualitative trends.

## Figures and Tables

**Table 1:** Fitted slope values and coefficients of determination for MSD linear regimes (1–5 ps) across temperatures for each component.

CaI_2_:CaCl_2_ (mol.%)	T/K	Linear Slope (m^2^/s)					
		Ca^2+^	R^2^	Cl^−^	R^2^	I^−^	R^2^
2:8	973	5.38 × 10^−9^	0.9993	8.88 × 10^−9^	0.9987	7.80 × 10^−9^	0.9964
	1023	8.61 × 10^−9^	0.9996	12.2 × 10^−9^	0.9987	9.42 × 10^−9^	0.9986
	1073	12.4 × 10^−9^	0.9989	15.8 × 10^−9^	0.9983	14.1 × 10^−9^	0.9961
	1123	16.4 × 10^−9^	0.9982	23.9 × 10^−9^	0.9996	18.7 × 10^−9^	0.9984
	1173	22.4 × 10^−9^	0.9996	31.4 × 10^−9^	0.9992	40.3 × 10^−9^	0.9971
3:7	973	11.6 × 10^−9^	0.9983	13.1 × 10^−9^	0.9983	10.8 × 10^−9^	0.9973
	1023	14.8 × 10^−9^	0.9994	16.1 × 10^−9^	0.9991	15.5 × 10^−9^	0.9978
	1073	17.3 × 10^−9^	0.9945	18.3 × 10^−9^	0.9989	25.3 × 10^−9^	0.9997
	1123	26.4 × 10^−9^	0.9981	28.7 × 10^−9^	0.9998	30.1 × 10^−9^	0.9994
	1173	22.0 × 10^−9^	0.9995	25.7 × 10^−9^	0.9986	28.7 × 10^−9^	0.9992

**Table 2:** Arrhenius parameters for ion self-diffusion at various temperatures and compositions in the CaCl_2_–CaI_2_ molten salt system (the data obtained at 1173 K were removed).

Ion	CaI_2_:CaCl_2_	Parameter		R^2^
	(mol.%)	E_a_ (kJ/mol)	D_0_ (m^2^/s)	
Ca^2+^	2:8	65.93	3.2517 × 10^−6^	0.9977
	3:7	46.52	0.5915 × 10^−6^	0.9436
Cl^−^	2:8	59.85	2.3508 × 10^−6^	0.9891
	3:7	43.84	0.4741 × 10^−6^	0.9086
I^−^	2:8	53.83	0.9678 × 10^−6^	0.9719
	3:7	64.02	5.0170 × 10^−6^	0.9793

**Table 3:** Radii for Ca^2+^, Cl^−^, and I^−^ ions at different coordination states (Å).

Ionic	Coordination	Ionic Radius
Ca^2+^	VI	1.00
	VII	1.06
	VIII	1.12
	IX	1.18
	X	1.23
	XII	1.34
Cl^−^	VI	1.81
I^−^	VI	2.20

## Data Availability

The original contributions presented in this study are included in the article/Supplementary Material. Further inquiries can be directed to the corresponding authors.

## Supplementary Materials

The following supporting information can be downloaded at: https://www.mdpi.com/article/10.3390/ma19101988/s1, Figure S1: Ion_1_-Ion_2_ coordination number.

## Abbreviations

The following abbreviations are used in this manuscript:

FPMD	first-principles molecular dynamics
CPMD	Car–Parrinello molecular dynamics
BOMD	Born–Oppenheimer molecular dynamics
DFT	Density functional theory
RDFs	Radial-distribution functions
PAW	Projected augmented wave
PBE	Perdew–Burke–Ernzerhof
LSF	Least squares fitting
CN	Coordination number
PMF	Potential of the mean force
MSD	Mean square displacement
