# Supplementary figures and images for: Microstructure and Transport Properties of CaCl2–CaI2 Molten Salt: A First-Principles Molecular Dynamics Study
Source: Materials (Basel). 2026 May 11;19(10):1988. doi: 10.3390/ma19101988 (PMC13209114; doi:10.3390/ma19101988)

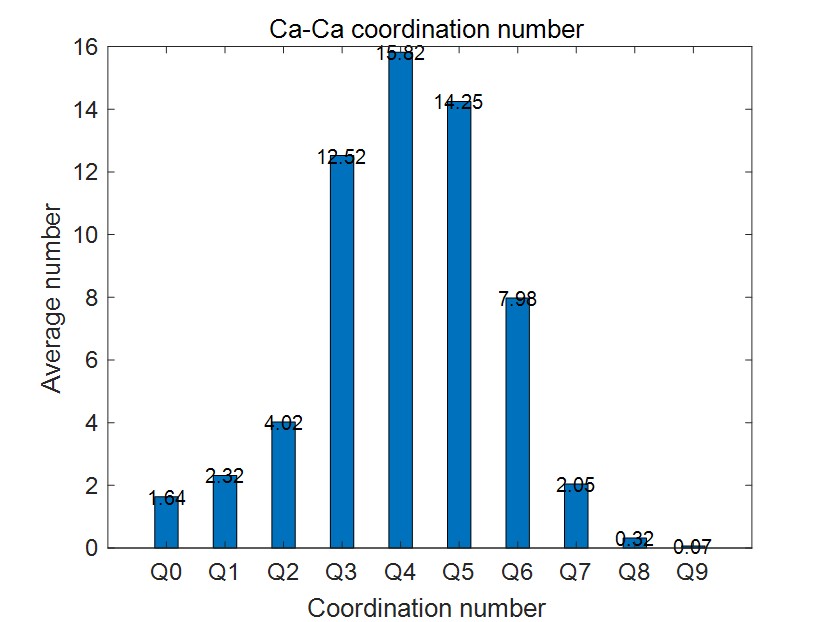

Supplement: Supplementary file 1 [file materials-19-01988-s001.zip › Figure S1/20molCaI2/1023K/CaCa.jpg]

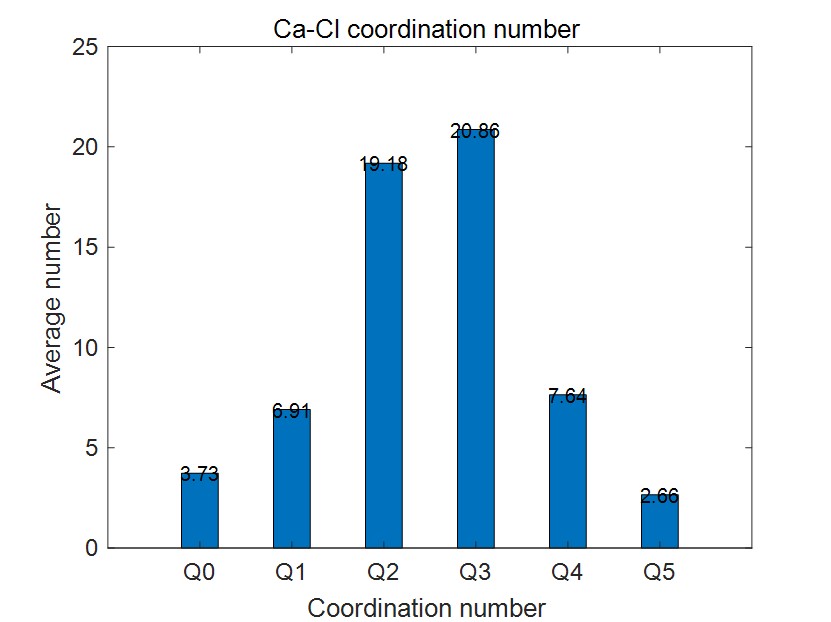

Supplement: Supplementary file 1 [file materials-19-01988-s001.zip › Figure S1/20molCaI2/1023K/CaCl.jpg]

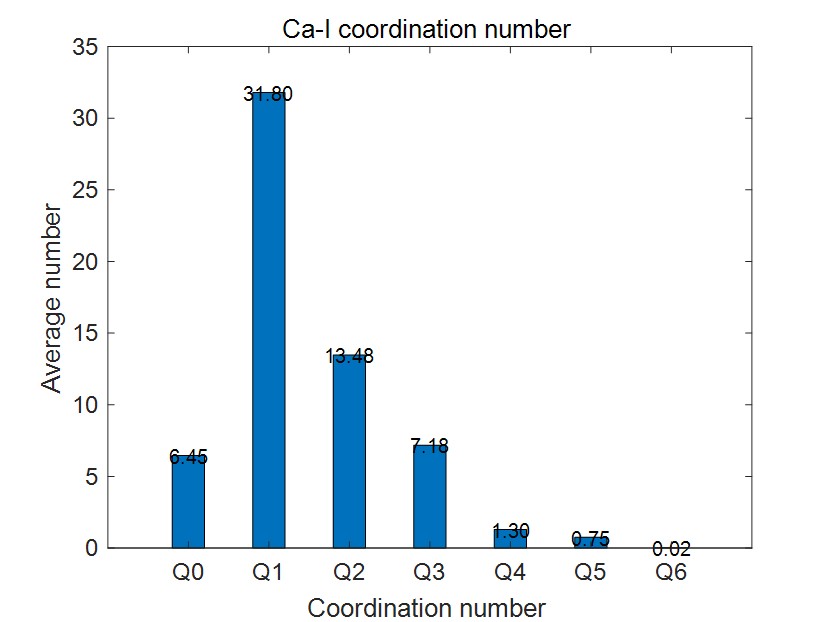

Supplement: Supplementary file 1 [file materials-19-01988-s001.zip › Figure S1/20molCaI2/1023K/CaI.jpg]

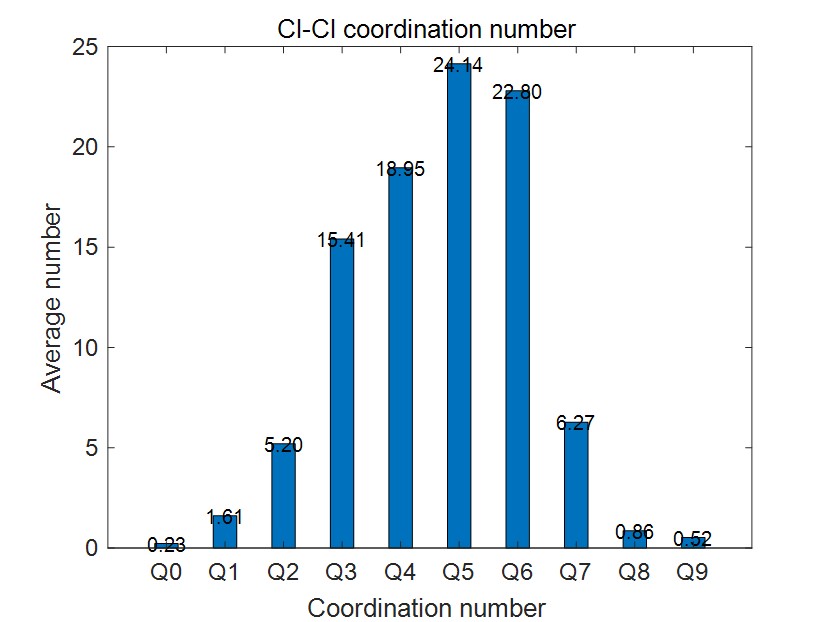

Supplement: Supplementary file 1 [file materials-19-01988-s001.zip › Figure S1/20molCaI2/1023K/ClCl.jpg]

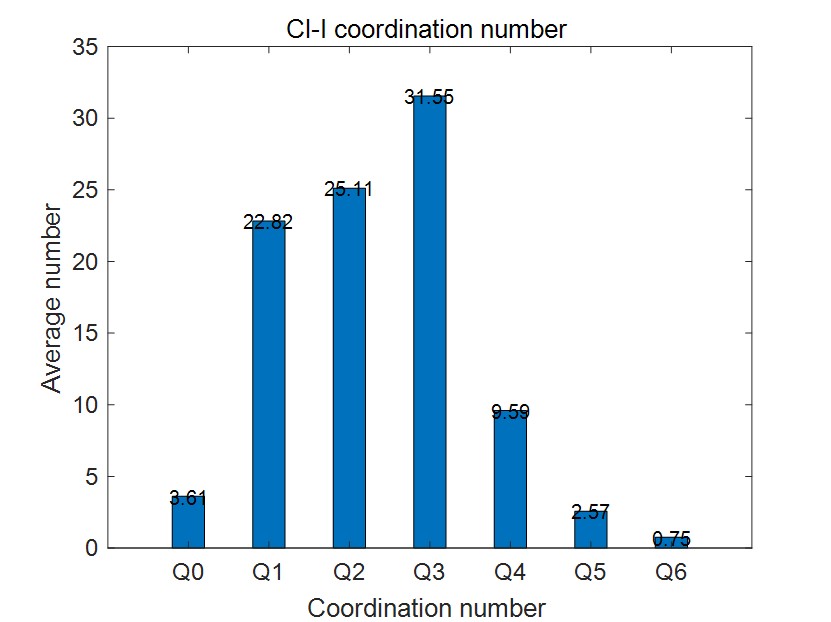

Supplement: Supplementary file 1 [file materials-19-01988-s001.zip › Figure S1/20molCaI2/1023K/ClI.jpg]

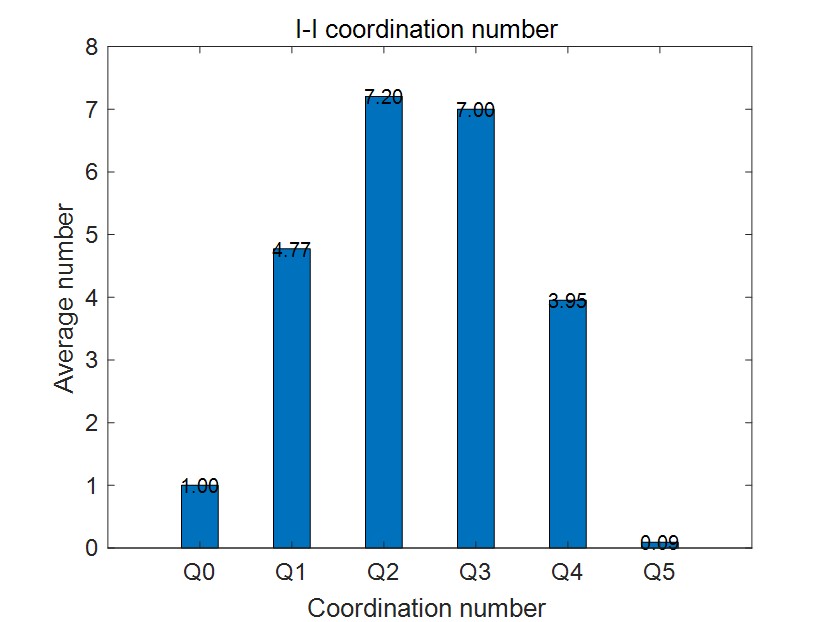

Supplement: Supplementary file 1 [file materials-19-01988-s001.zip › Figure S1/20molCaI2/1023K/II.jpg]

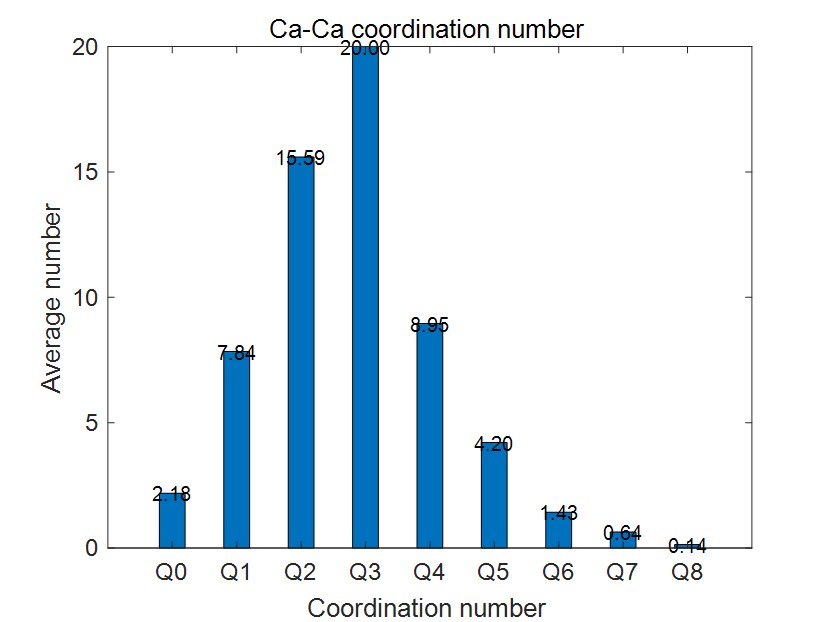

Supplement: Supplementary file 1 [file materials-19-01988-s001.zip › Figure S1/20molCaI2/1073K/CaCa.jpg]

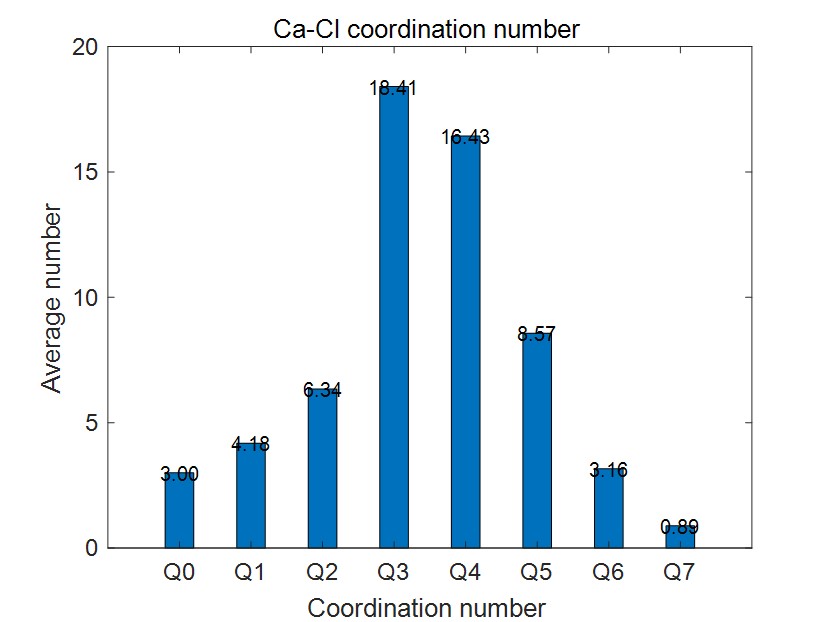

Supplement: Supplementary file 1 [file materials-19-01988-s001.zip › Figure S1/20molCaI2/1073K/CaCl.jpg]

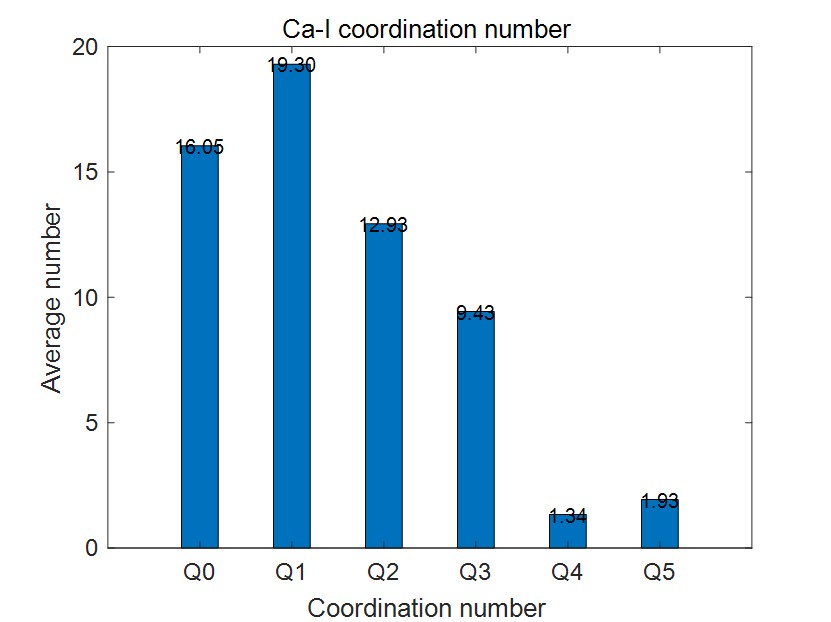

Supplement: Supplementary file 1 [file materials-19-01988-s001.zip › Figure S1/20molCaI2/1073K/CaI.jpg]

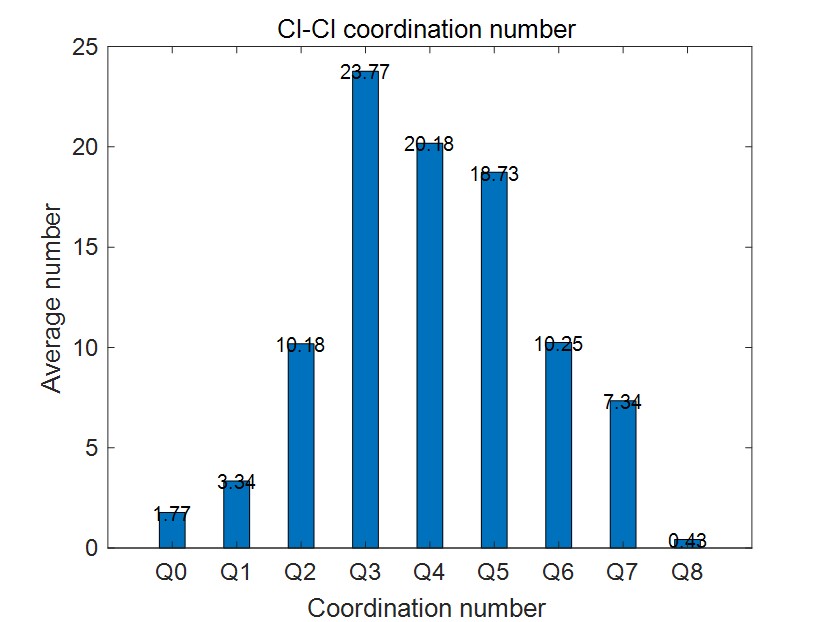

Supplement: Supplementary file 1 [file materials-19-01988-s001.zip › Figure S1/20molCaI2/1073K/ClCl.jpg]

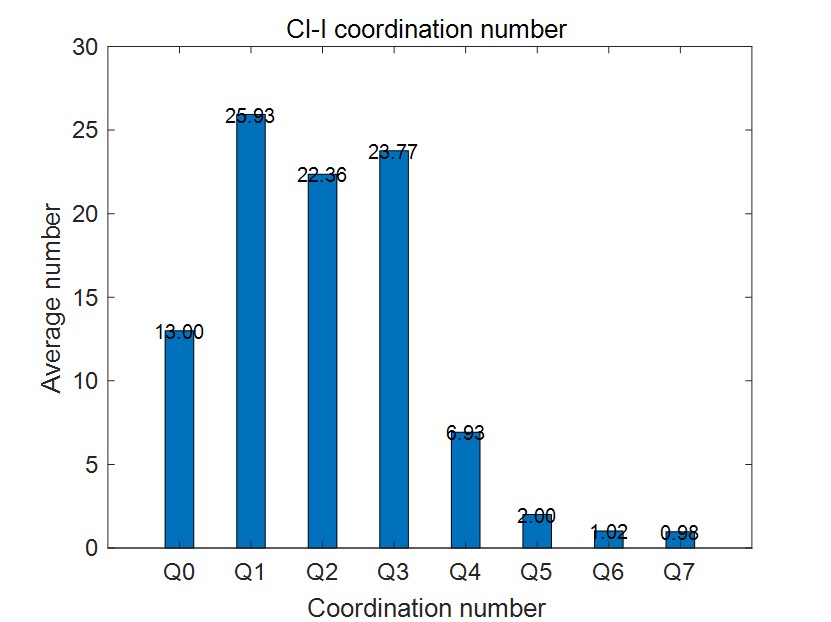

Supplement: Supplementary file 1 [file materials-19-01988-s001.zip › Figure S1/20molCaI2/1073K/ClI.jpg]

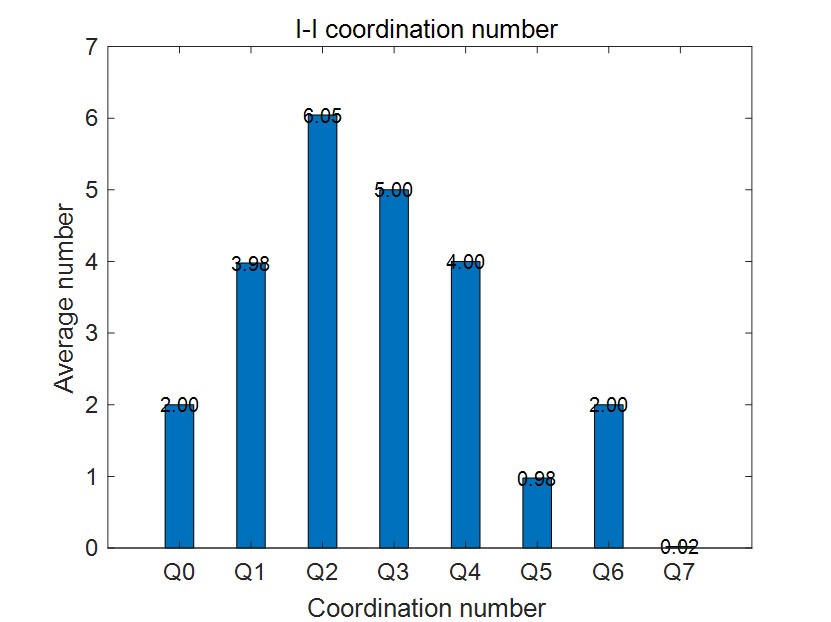

Supplement: Supplementary file 1 [file materials-19-01988-s001.zip › Figure S1/20molCaI2/1073K/II.jpg]

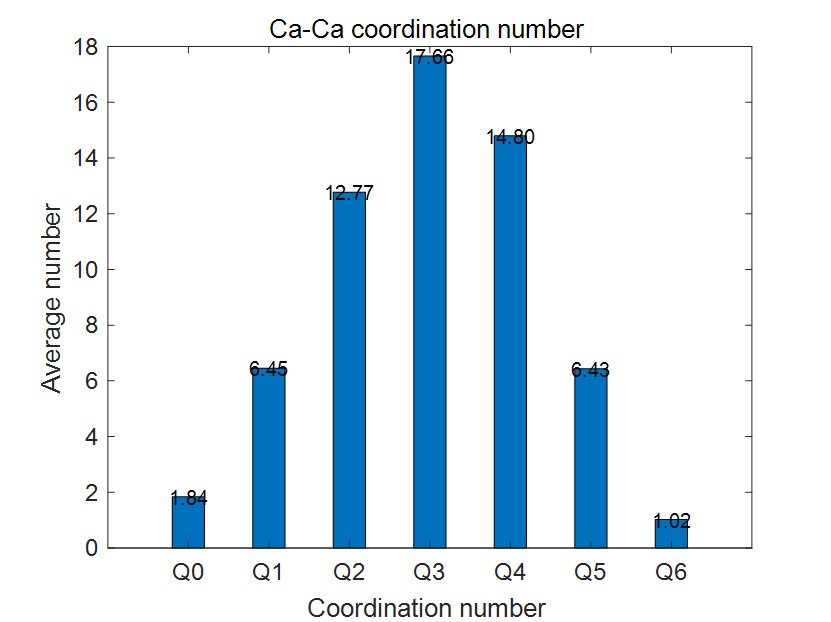

Supplement: Supplementary file 1 [file materials-19-01988-s001.zip › Figure S1/20molCaI2/1123K/CaCa.jpg]

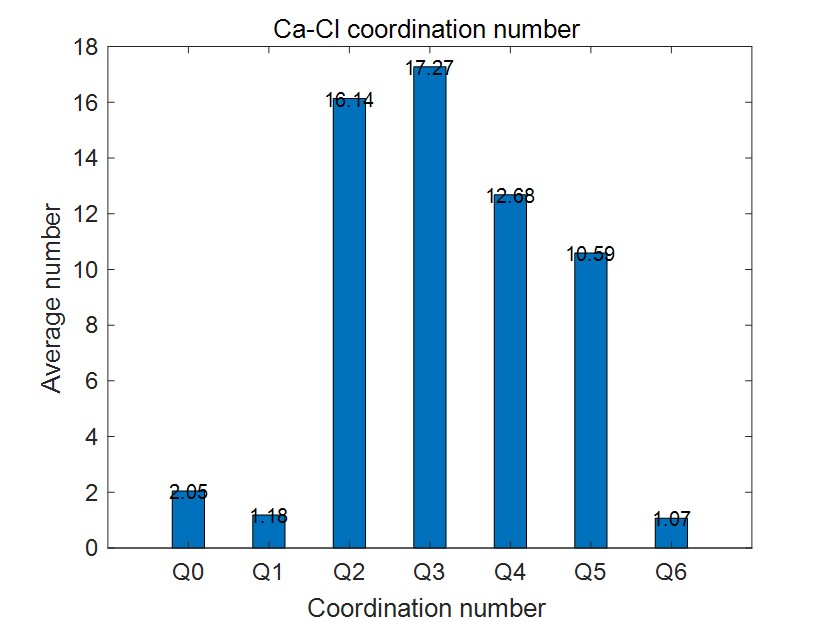

Supplement: Supplementary file 1 [file materials-19-01988-s001.zip › Figure S1/20molCaI2/1123K/CaCl.jpg]

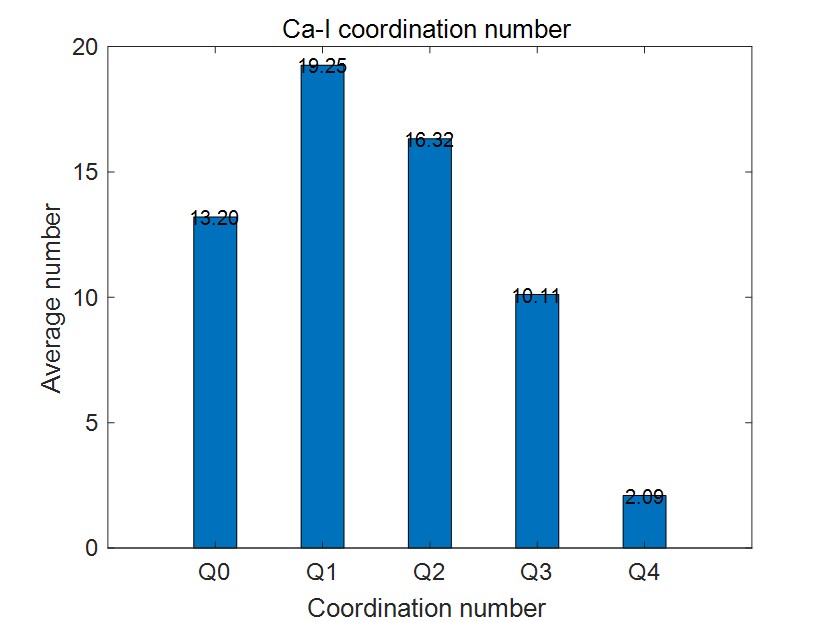

Supplement: Supplementary file 1 [file materials-19-01988-s001.zip › Figure S1/20molCaI2/1123K/CaI.jpg]

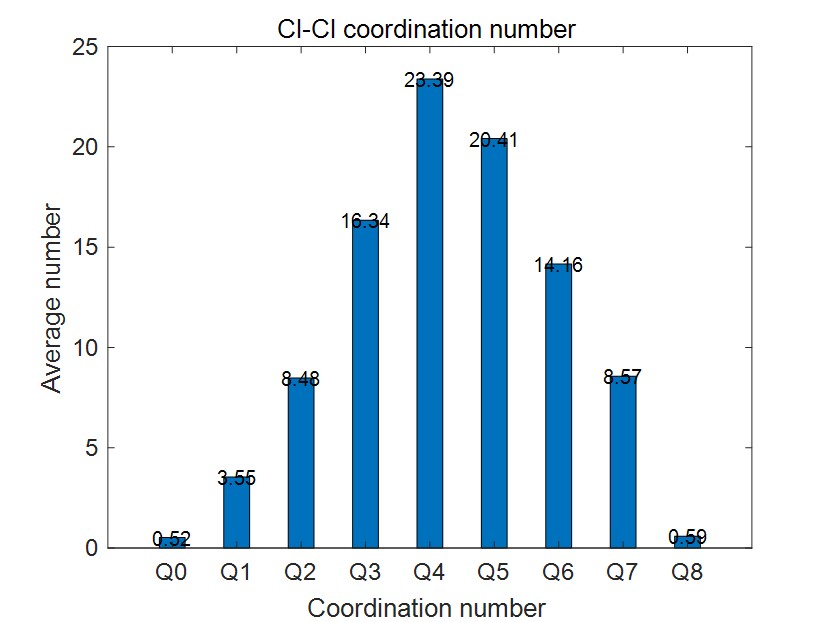

Supplement: Supplementary file 1 [file materials-19-01988-s001.zip › Figure S1/20molCaI2/1123K/ClCl.jpg]

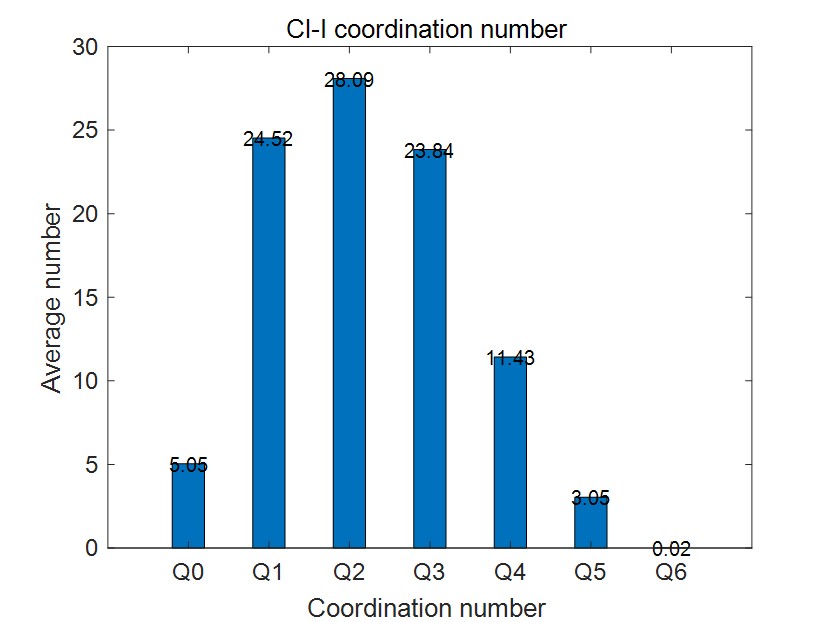

Supplement: Supplementary file 1 [file materials-19-01988-s001.zip › Figure S1/20molCaI2/1123K/ClI.jpg]

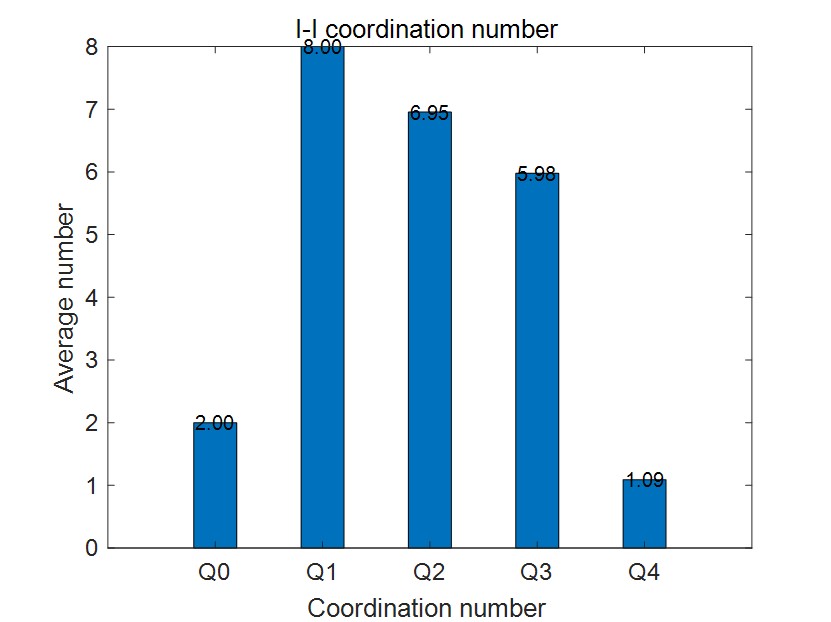

Supplement: Supplementary file 1 [file materials-19-01988-s001.zip › Figure S1/20molCaI2/1123K/II.jpg]

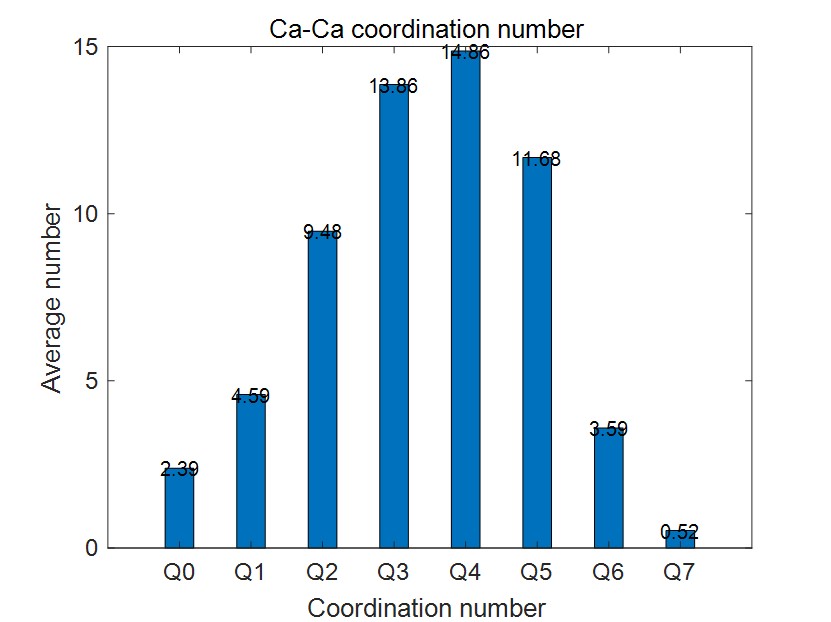

Supplement: Supplementary file 1 [file materials-19-01988-s001.zip › Figure S1/20molCaI2/1173K/CaCa.jpg]

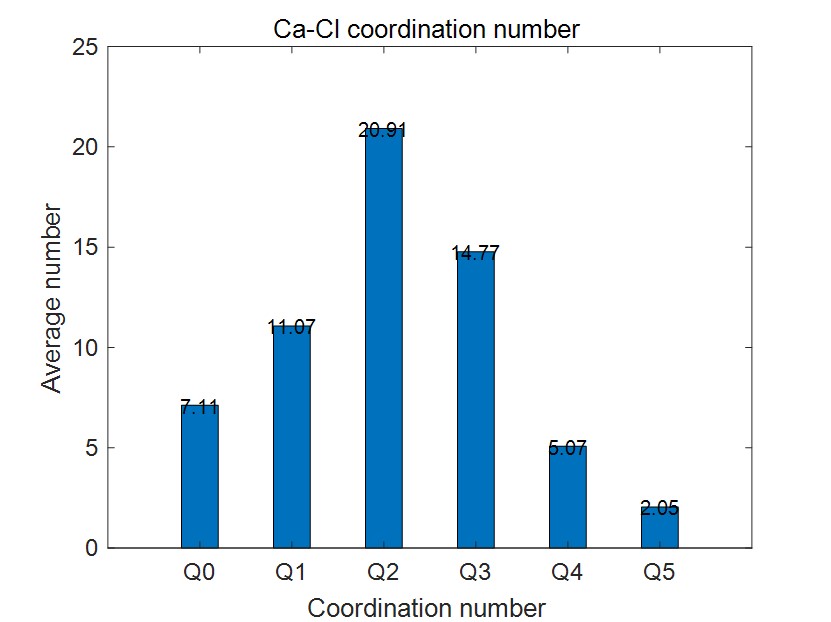

Supplement: Supplementary file 1 [file materials-19-01988-s001.zip › Figure S1/20molCaI2/1173K/CaCl.jpg]

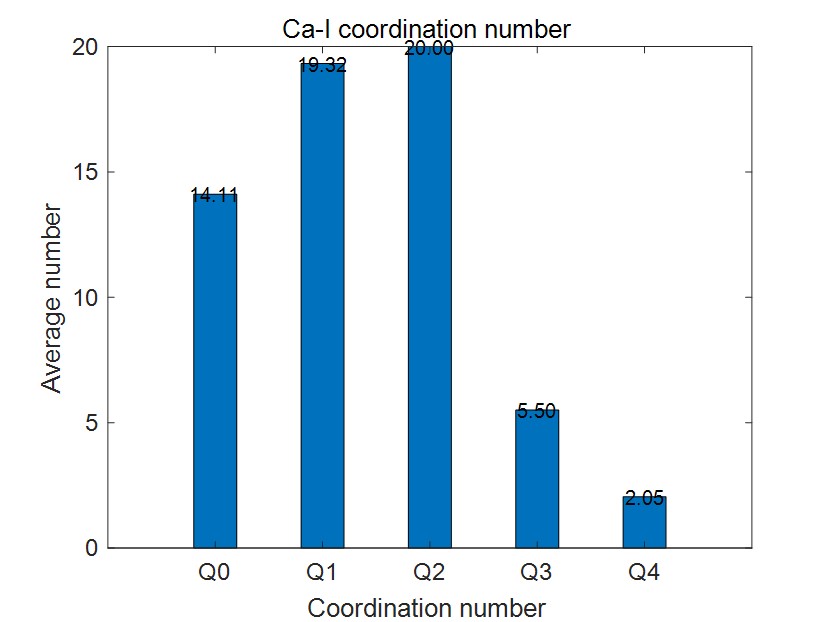

Supplement: Supplementary file 1 [file materials-19-01988-s001.zip › Figure S1/20molCaI2/1173K/CaI.jpg]

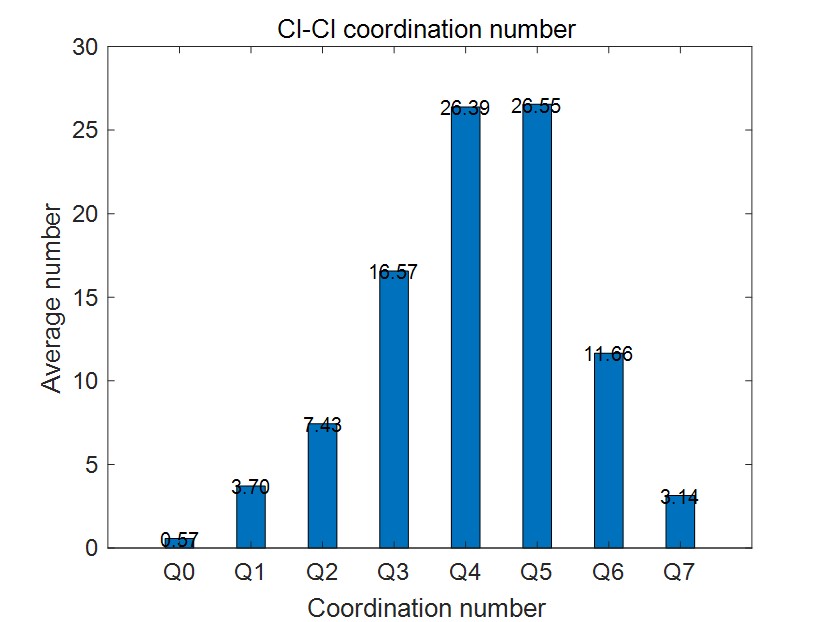

Supplement: Supplementary file 1 [file materials-19-01988-s001.zip › Figure S1/20molCaI2/1173K/ClCl.jpg]

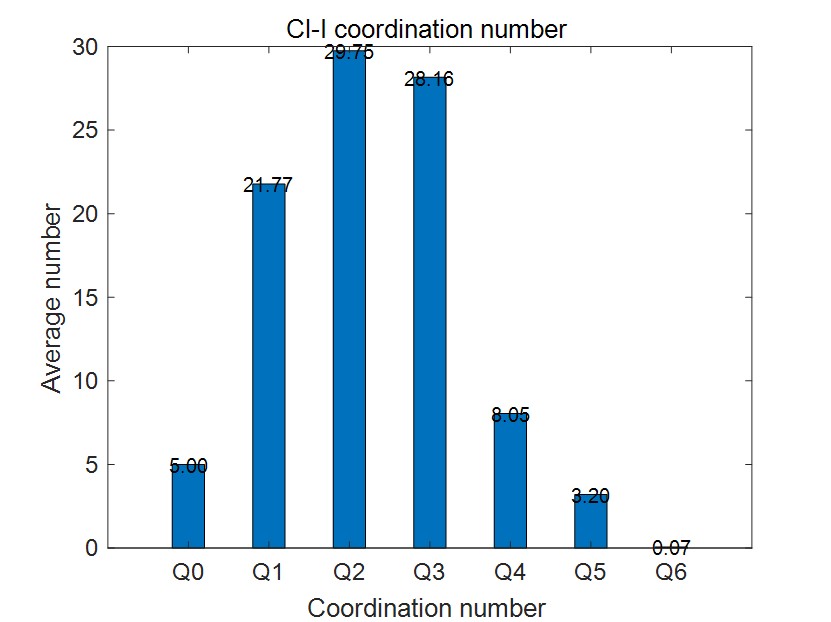

Supplement: Supplementary file 1 [file materials-19-01988-s001.zip › Figure S1/20molCaI2/1173K/ClI.jpg]

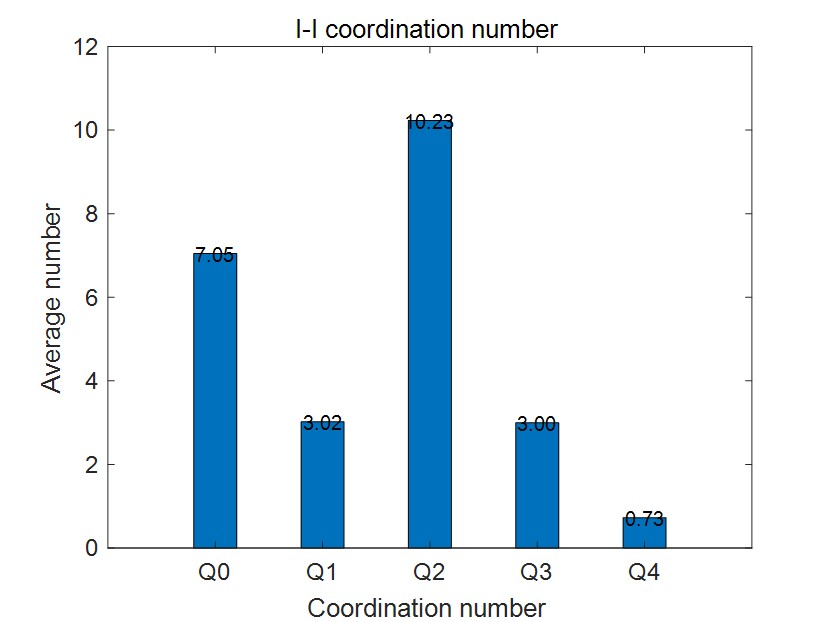

Supplement: Supplementary file 1 [file materials-19-01988-s001.zip › Figure S1/20molCaI2/1173K/II.jpg]

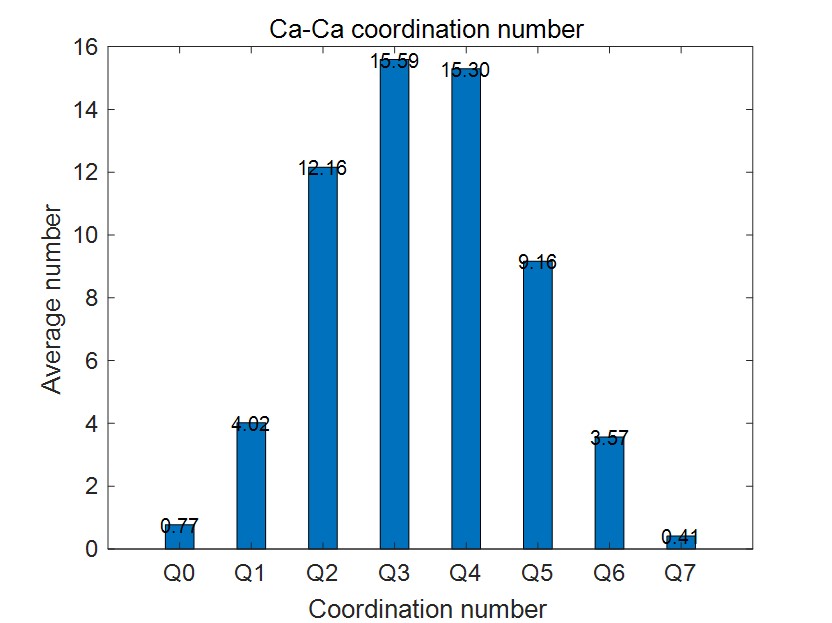

Supplement: Supplementary file 1 [file materials-19-01988-s001.zip › Figure S1/20molCaI2/973K/CaCa.jpg]

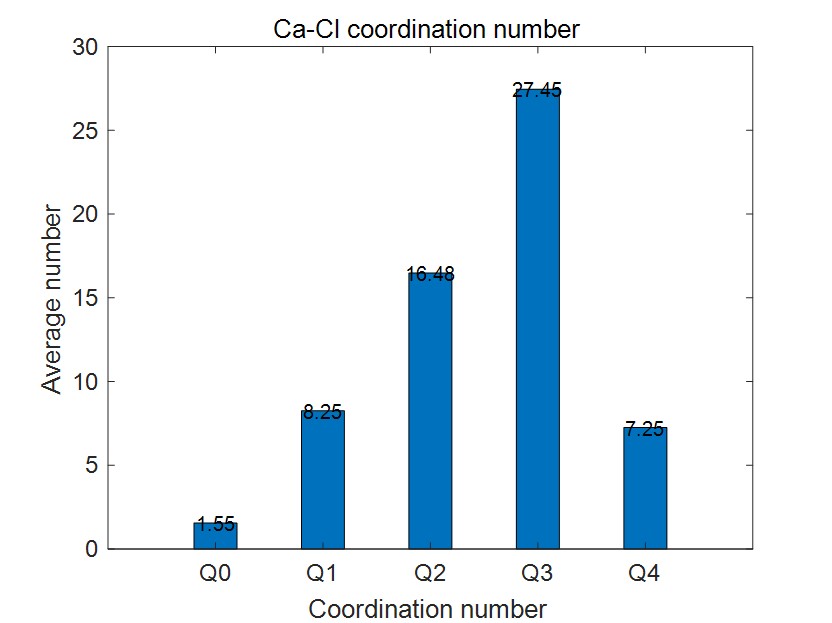

Supplement: Supplementary file 1 [file materials-19-01988-s001.zip › Figure S1/20molCaI2/973K/CaCl.jpg]

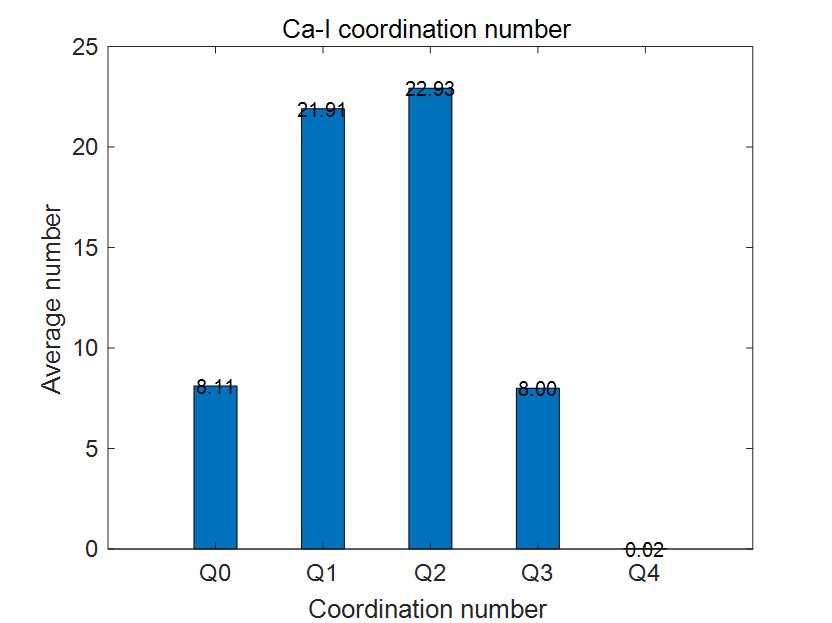

Supplement: Supplementary file 1 [file materials-19-01988-s001.zip › Figure S1/20molCaI2/973K/CaI.jpg]

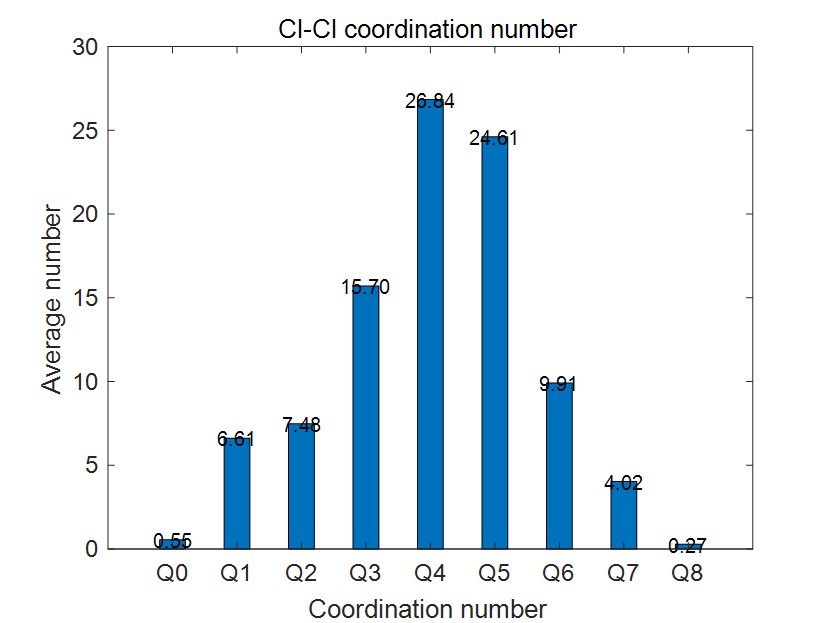

Supplement: Supplementary file 1 [file materials-19-01988-s001.zip › Figure S1/20molCaI2/973K/ClCl.jpg]

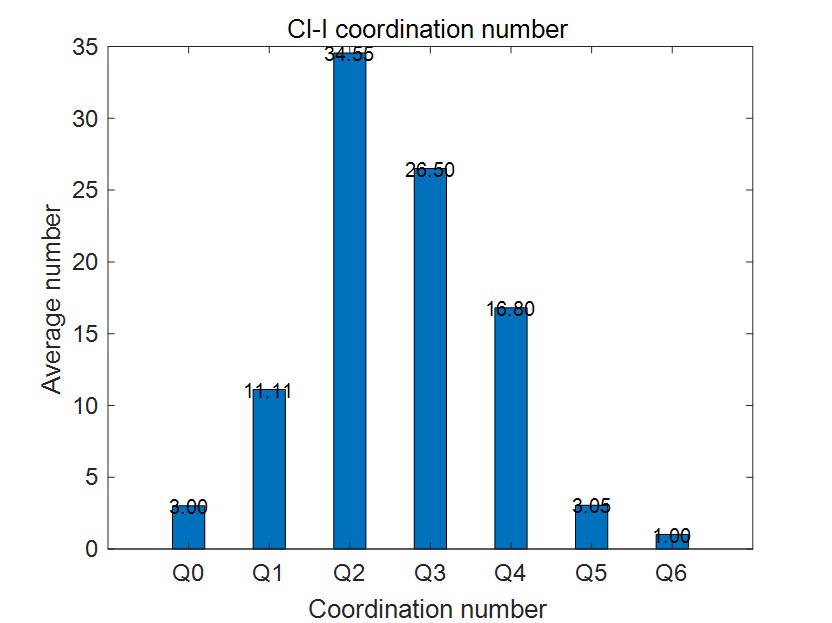

Supplement: Supplementary file 1 [file materials-19-01988-s001.zip › Figure S1/20molCaI2/973K/ClI.jpg]

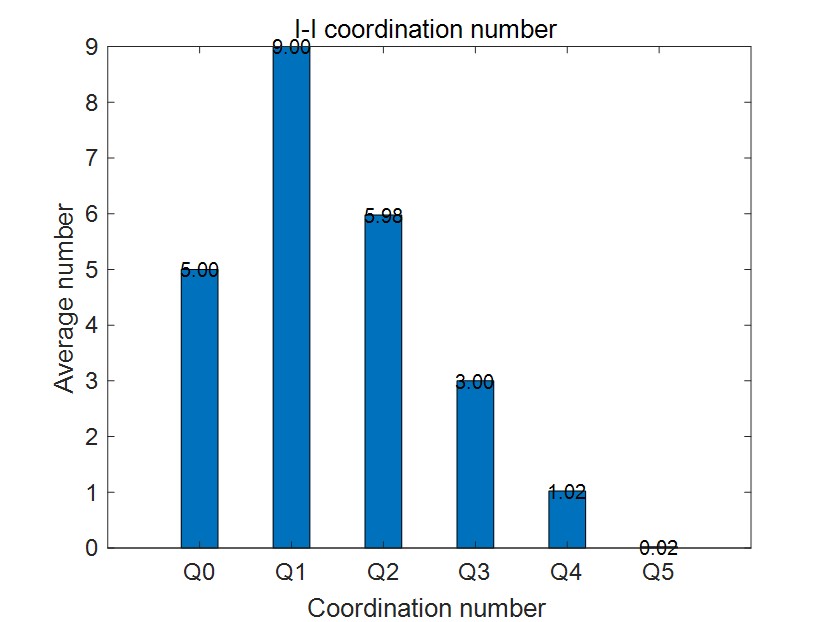

Supplement: Supplementary file 1 [file materials-19-01988-s001.zip › Figure S1/20molCaI2/973K/II.jpg]

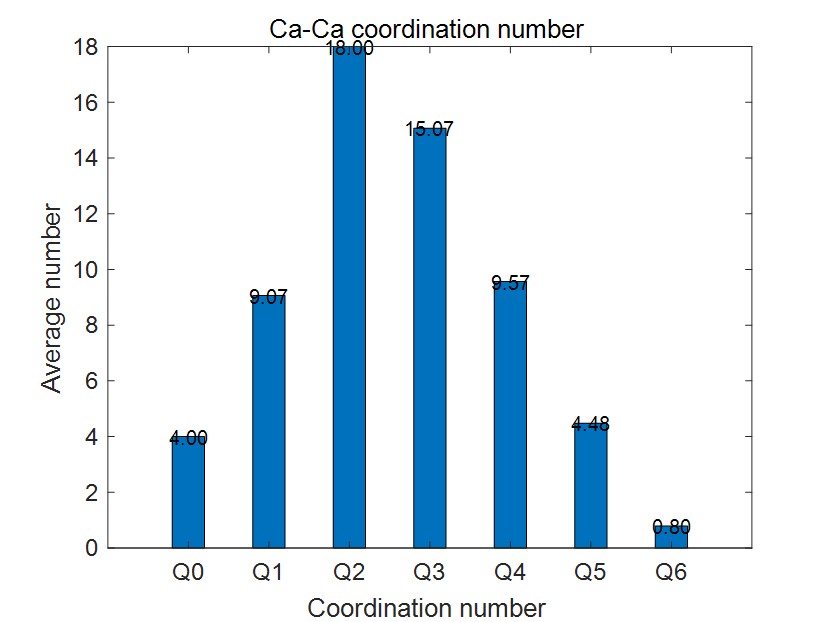

Supplement: Supplementary file 1 [file materials-19-01988-s001.zip › Figure S1/30molCaI2/1023K/CaCa.jpg]

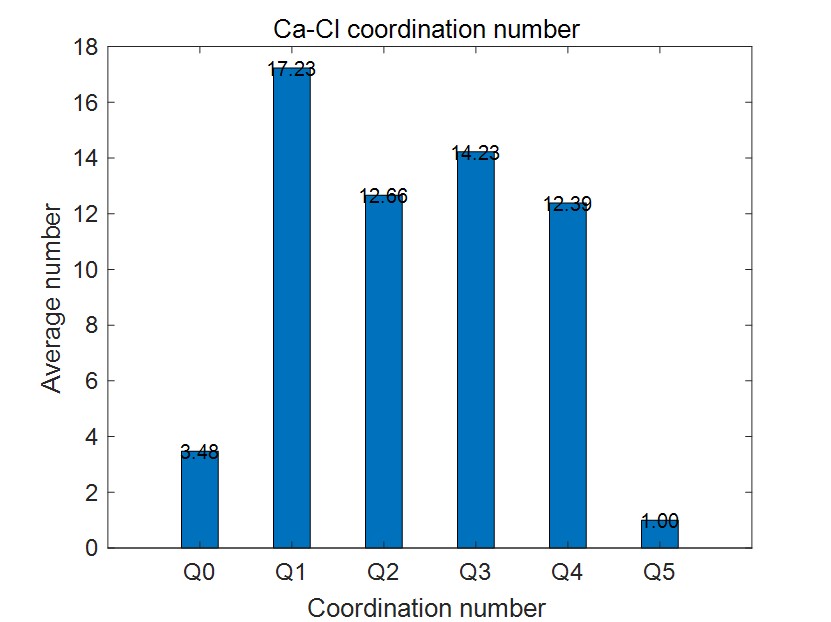

Supplement: Supplementary file 1 [file materials-19-01988-s001.zip › Figure S1/30molCaI2/1023K/CaCl.jpg]

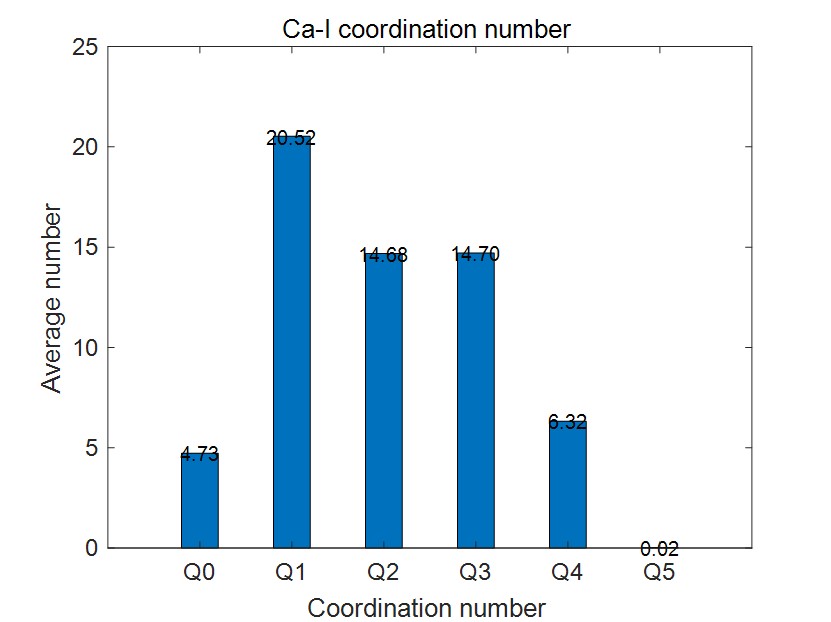

Supplement: Supplementary file 1 [file materials-19-01988-s001.zip › Figure S1/30molCaI2/1023K/CaI.jpg]

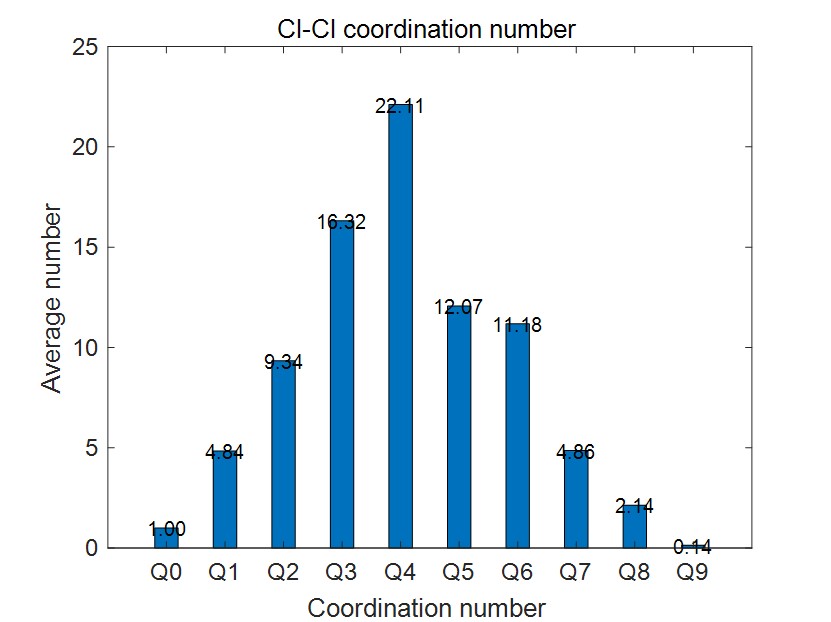

Supplement: Supplementary file 1 [file materials-19-01988-s001.zip › Figure S1/30molCaI2/1023K/ClCl.jpg]

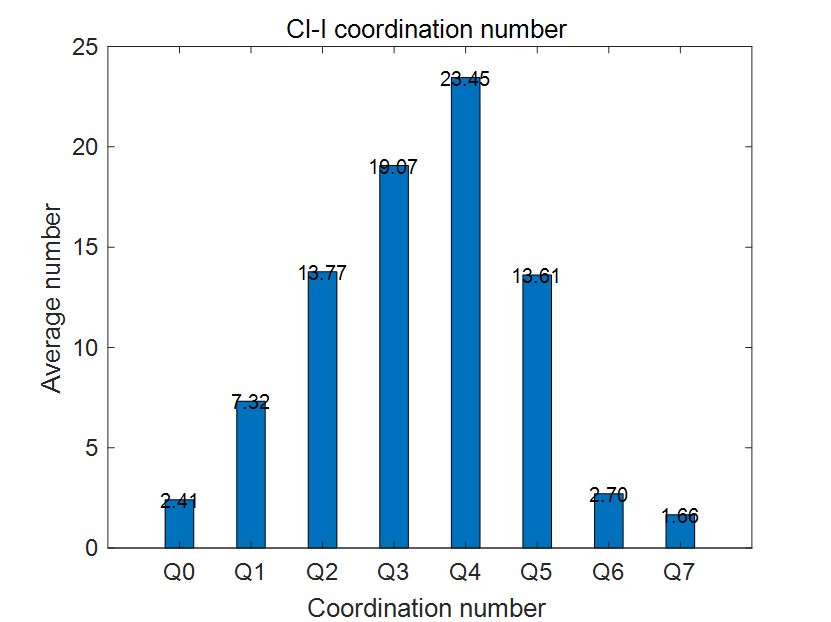

Supplement: Supplementary file 1 [file materials-19-01988-s001.zip › Figure S1/30molCaI2/1023K/ClI.jpg]

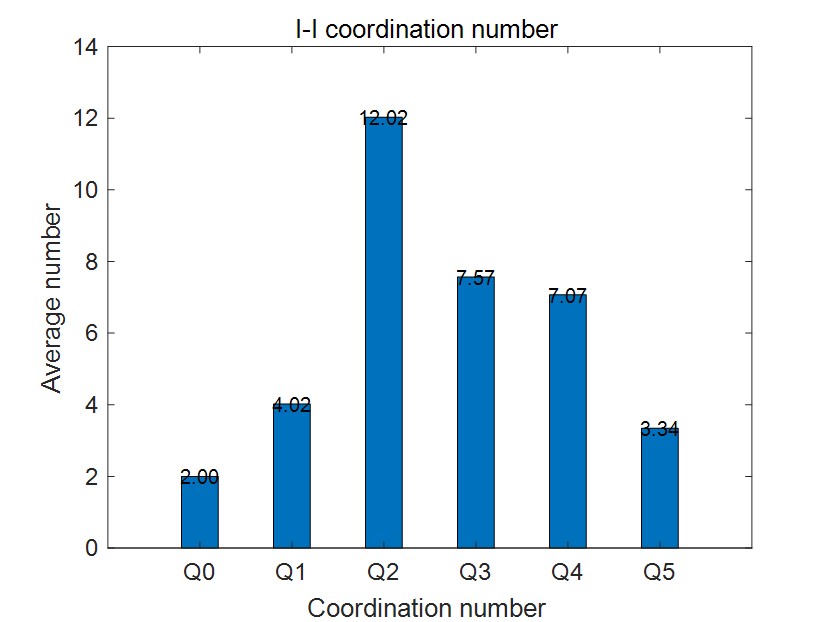

Supplement: Supplementary file 1 [file materials-19-01988-s001.zip › Figure S1/30molCaI2/1023K/II.jpg]

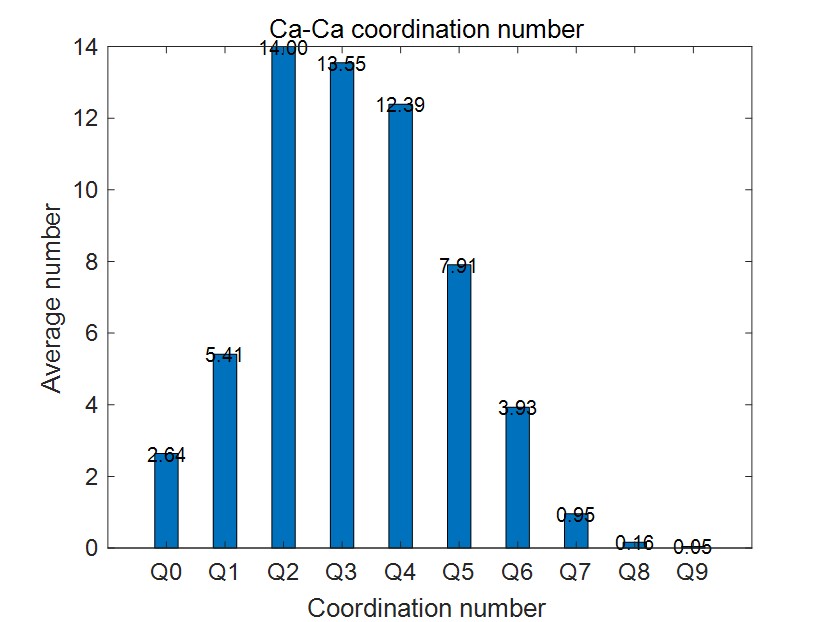

Supplement: Supplementary file 1 [file materials-19-01988-s001.zip › Figure S1/30molCaI2/1073K/CaCa.jpg]

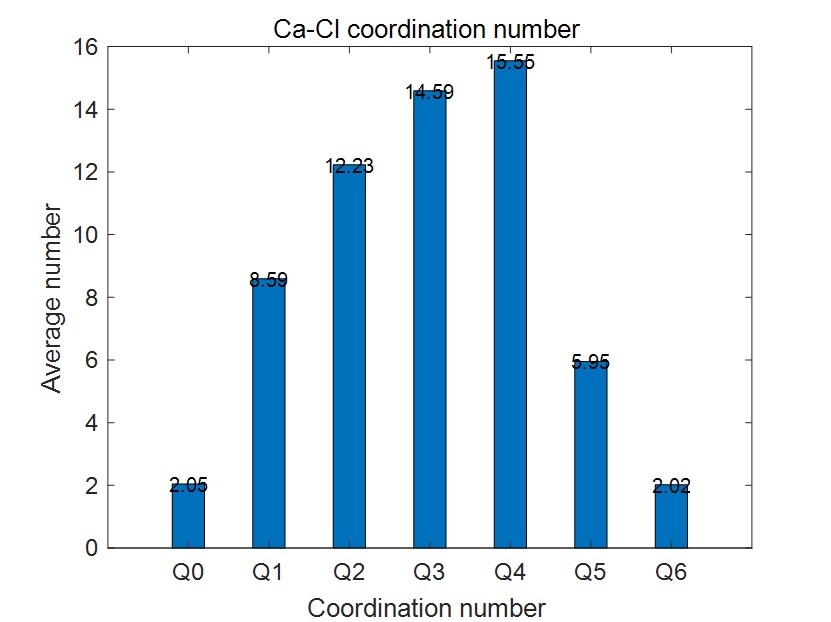

Supplement: Supplementary file 1 [file materials-19-01988-s001.zip › Figure S1/30molCaI2/1073K/CaCl.jpg]

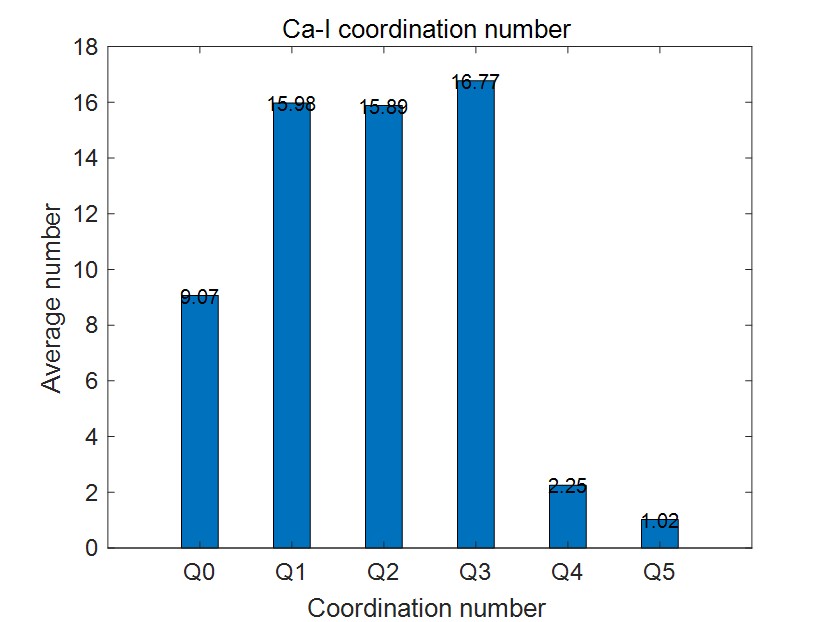

Supplement: Supplementary file 1 [file materials-19-01988-s001.zip › Figure S1/30molCaI2/1073K/CaI.jpg]

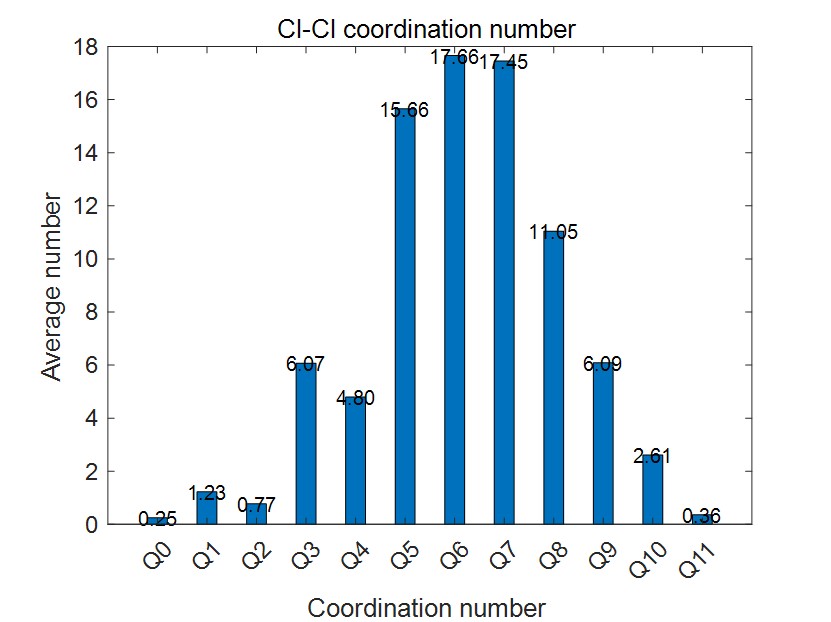

Supplement: Supplementary file 1 [file materials-19-01988-s001.zip › Figure S1/30molCaI2/1073K/ClCl.jpg]

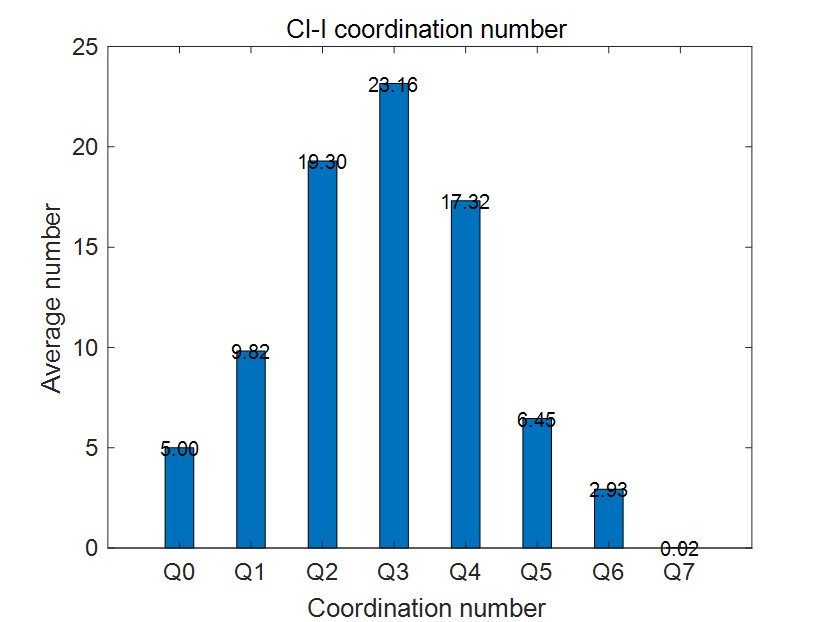

Supplement: Supplementary file 1 [file materials-19-01988-s001.zip › Figure S1/30molCaI2/1073K/ClI.jpg]

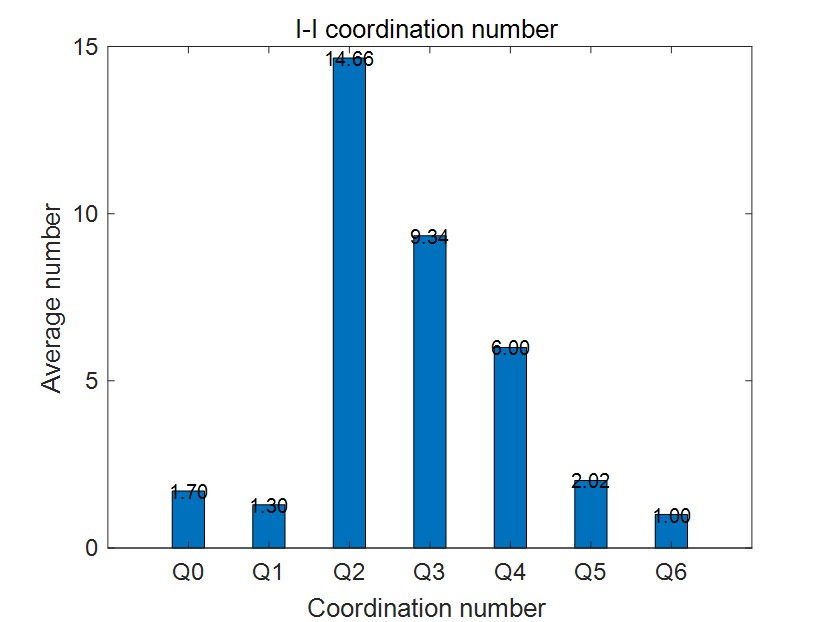

Supplement: Supplementary file 1 [file materials-19-01988-s001.zip › Figure S1/30molCaI2/1073K/II.jpg]

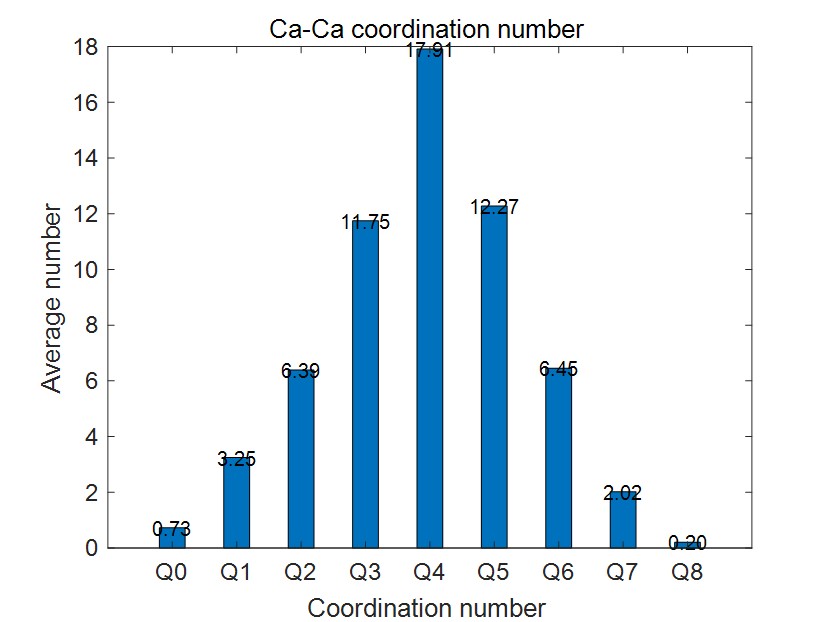

Supplement: Supplementary file 1 [file materials-19-01988-s001.zip › Figure S1/30molCaI2/1123K/CaCa.jpg]

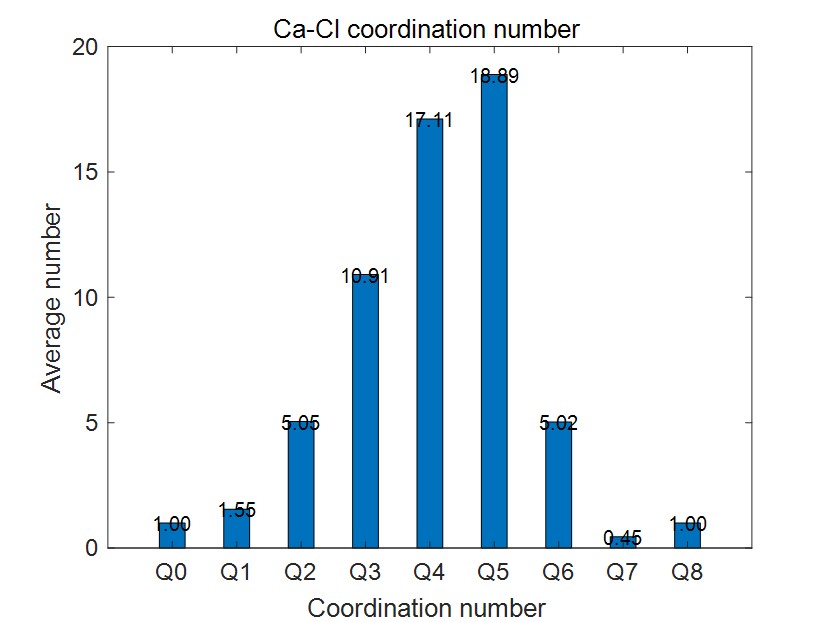

Supplement: Supplementary file 1 [file materials-19-01988-s001.zip › Figure S1/30molCaI2/1123K/CaCl.jpg]

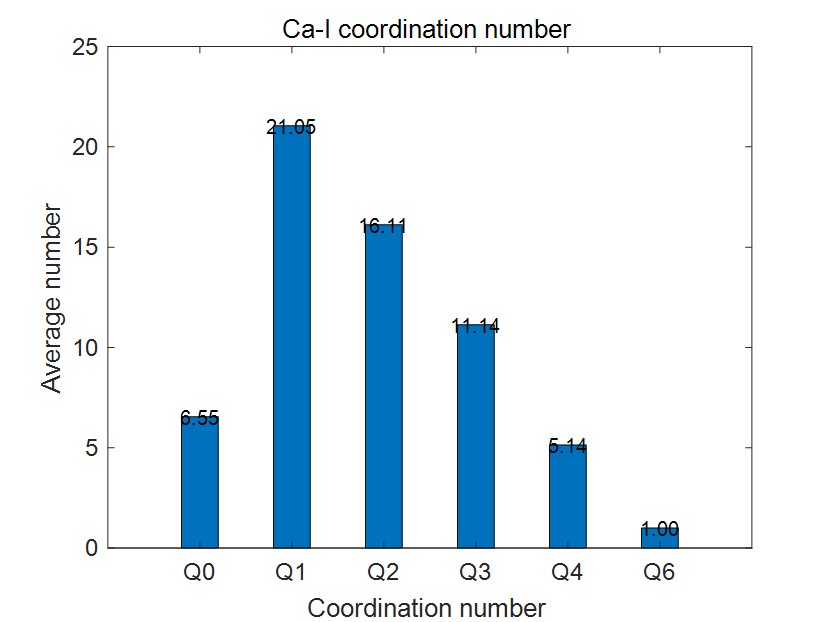

Supplement: Supplementary file 1 [file materials-19-01988-s001.zip › Figure S1/30molCaI2/1123K/CaI.jpg]

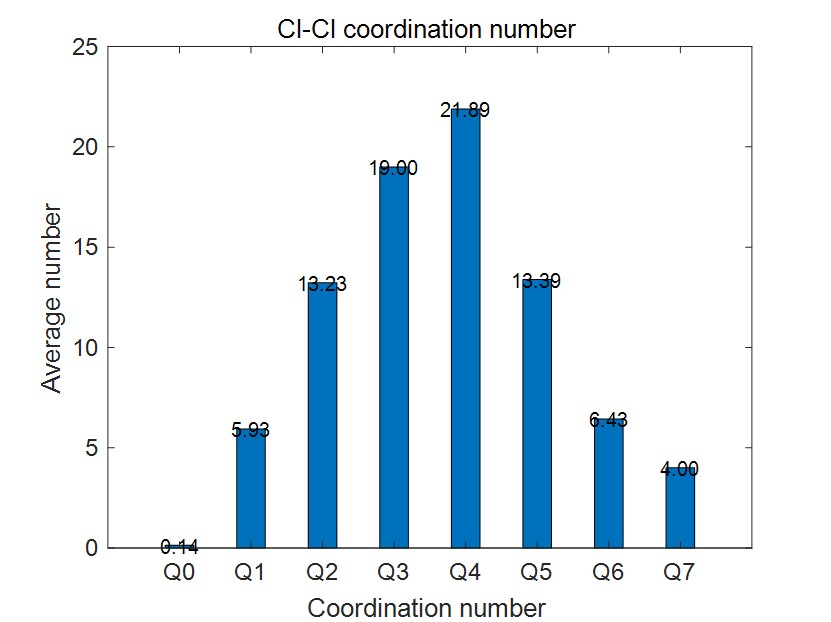

Supplement: Supplementary file 1 [file materials-19-01988-s001.zip › Figure S1/30molCaI2/1123K/ClCl.jpg]

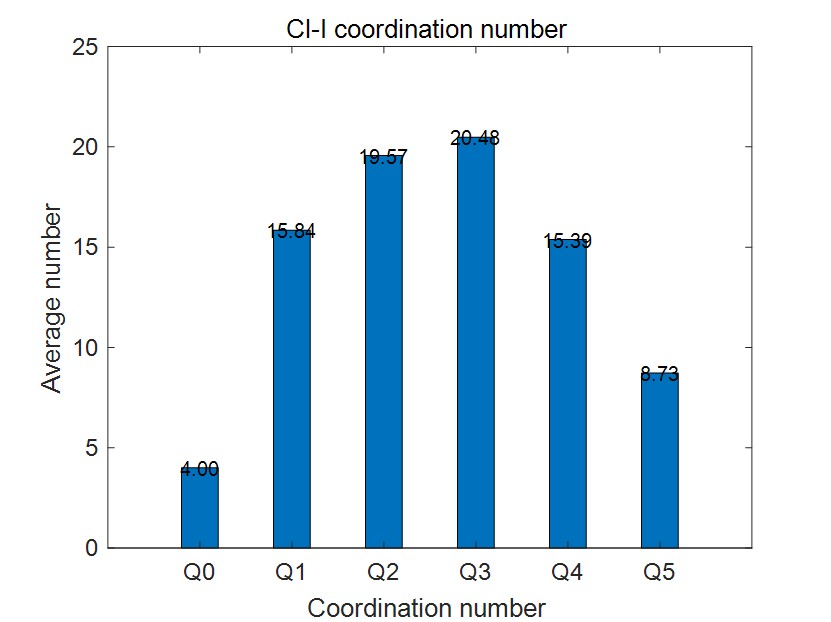

Supplement: Supplementary file 1 [file materials-19-01988-s001.zip › Figure S1/30molCaI2/1123K/ClI.jpg]

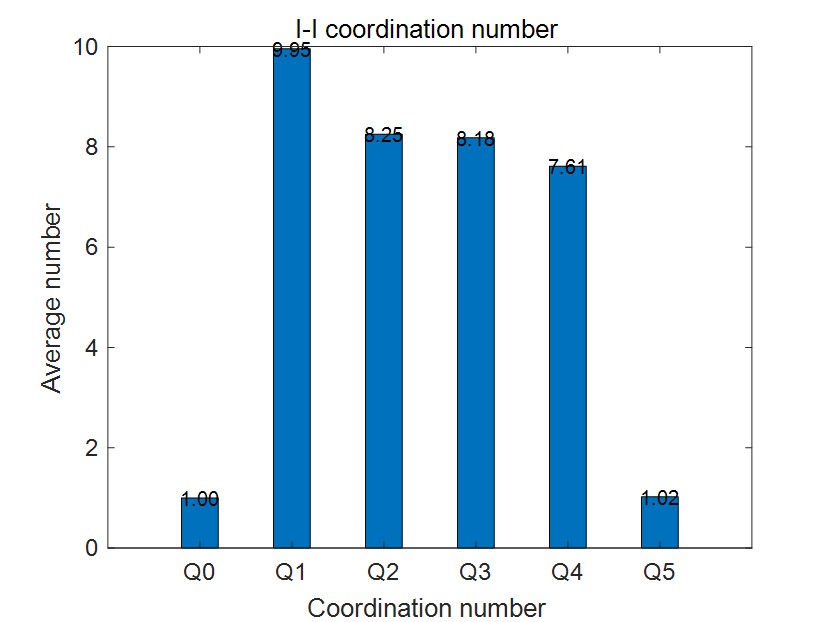

Supplement: Supplementary file 1 [file materials-19-01988-s001.zip › Figure S1/30molCaI2/1123K/II.jpg]

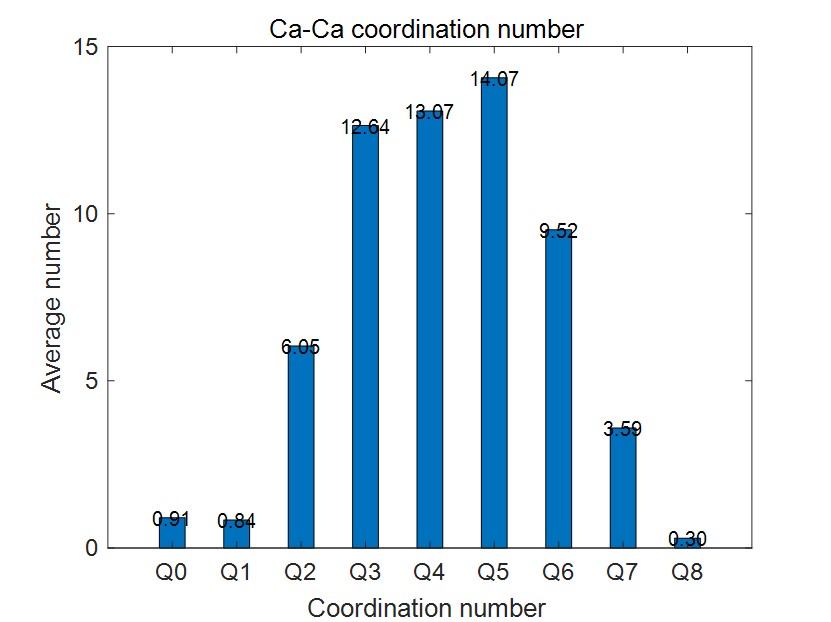

Supplement: Supplementary file 1 [file materials-19-01988-s001.zip › Figure S1/30molCaI2/1173K/CaCa.jpg]

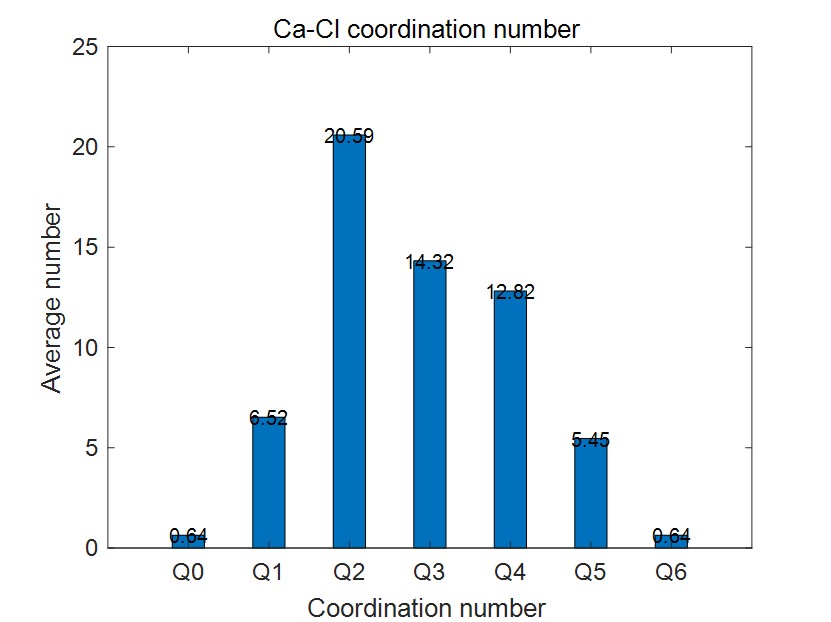

Supplement: Supplementary file 1 [file materials-19-01988-s001.zip › Figure S1/30molCaI2/1173K/CaCl.jpg]

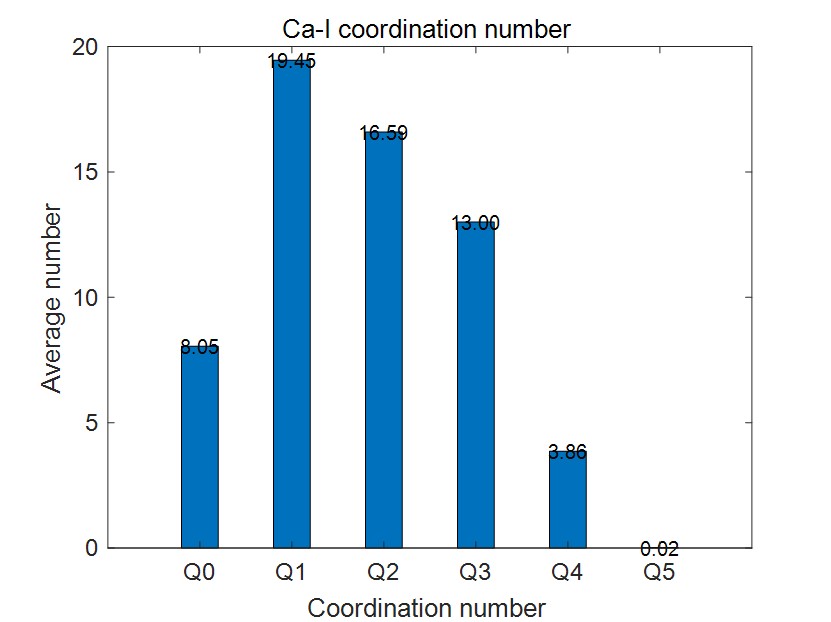

Supplement: Supplementary file 1 [file materials-19-01988-s001.zip › Figure S1/30molCaI2/1173K/CaI.jpg]

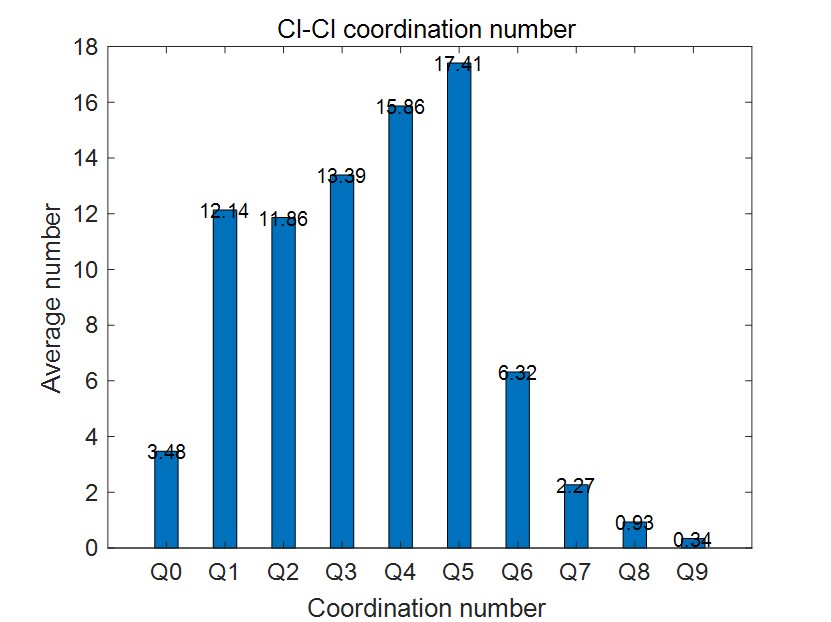

Supplement: Supplementary file 1 [file materials-19-01988-s001.zip › Figure S1/30molCaI2/1173K/ClCl.jpg]

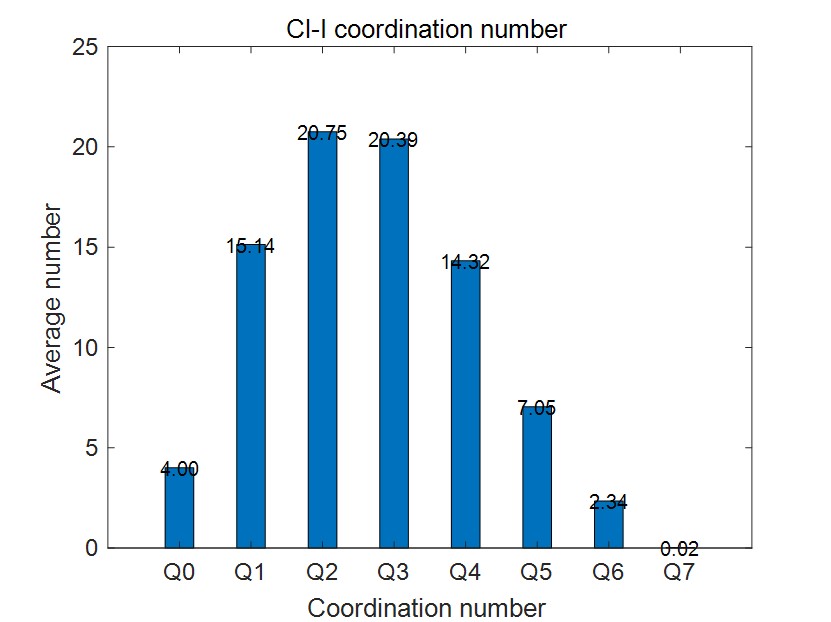

Supplement: Supplementary file 1 [file materials-19-01988-s001.zip › Figure S1/30molCaI2/1173K/ClI.jpg]

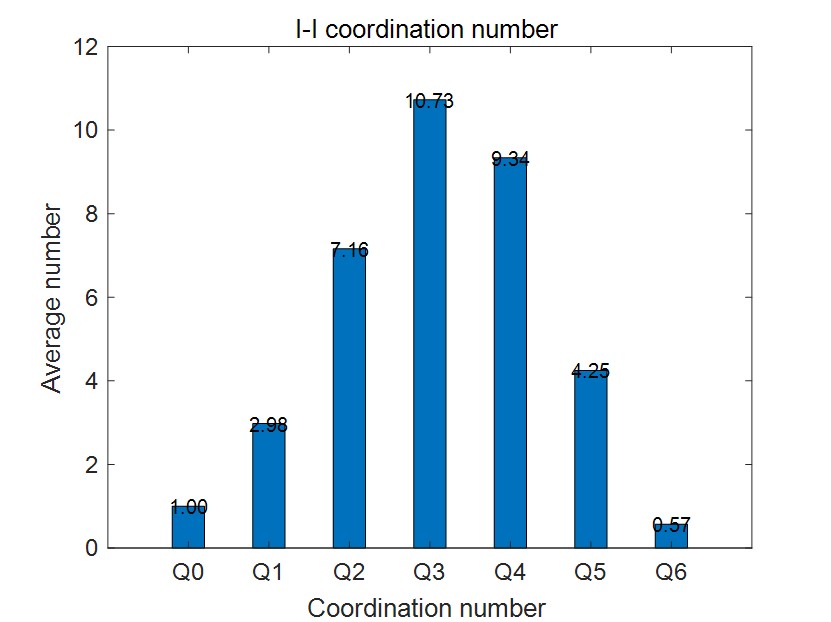

Supplement: Supplementary file 1 [file materials-19-01988-s001.zip › Figure S1/30molCaI2/1173K/II.jpg]

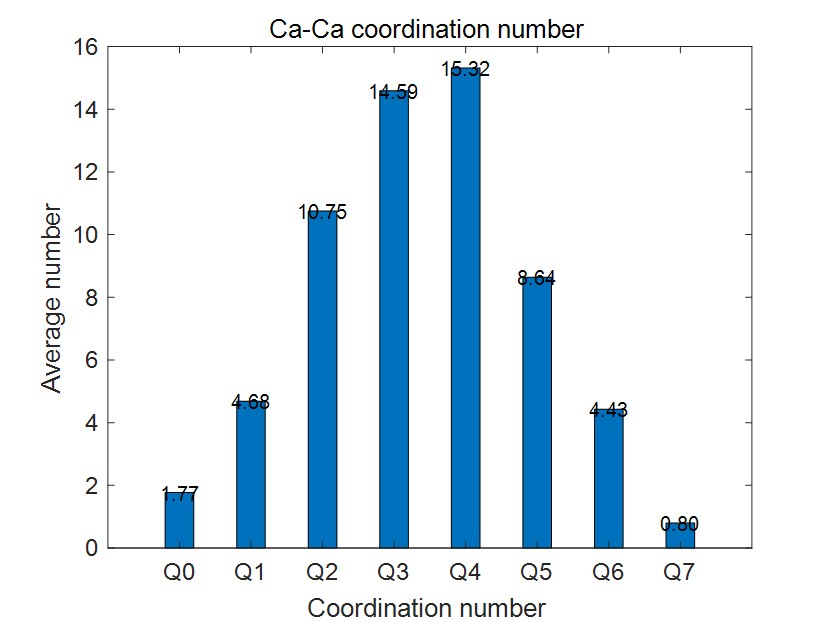

Supplement: Supplementary file 1 [file materials-19-01988-s001.zip › Figure S1/30molCaI2/973K/CaCa.jpg]

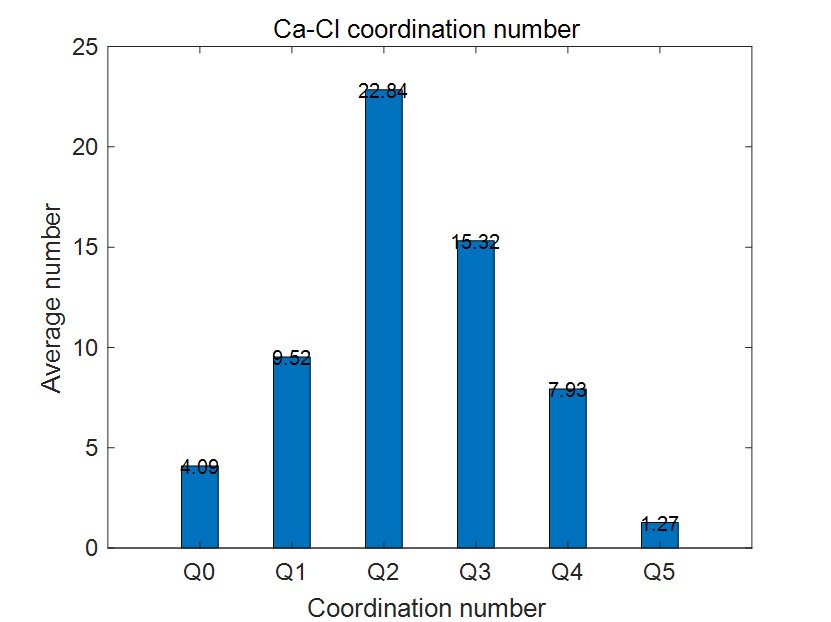

Supplement: Supplementary file 1 [file materials-19-01988-s001.zip › Figure S1/30molCaI2/973K/CaCl.jpg]

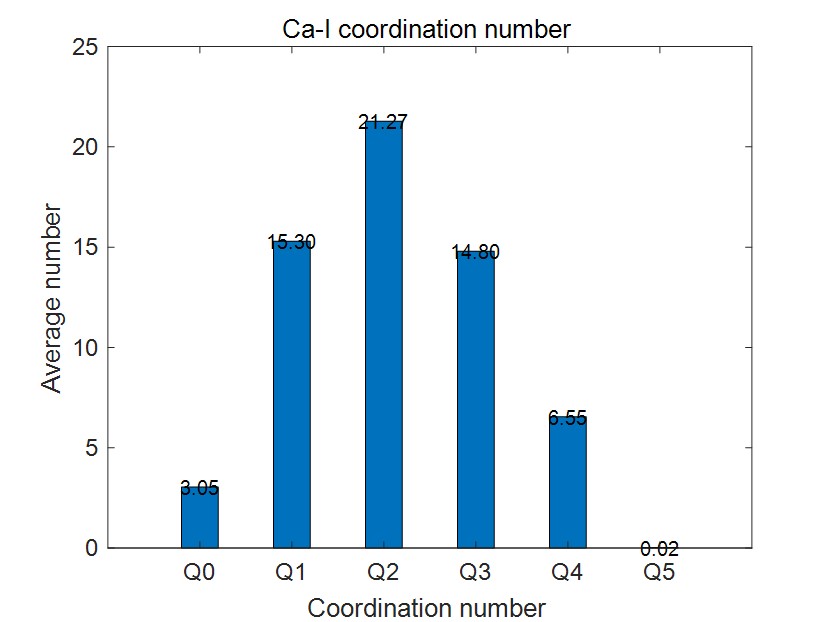

Supplement: Supplementary file 1 [file materials-19-01988-s001.zip › Figure S1/30molCaI2/973K/CaI.jpg]

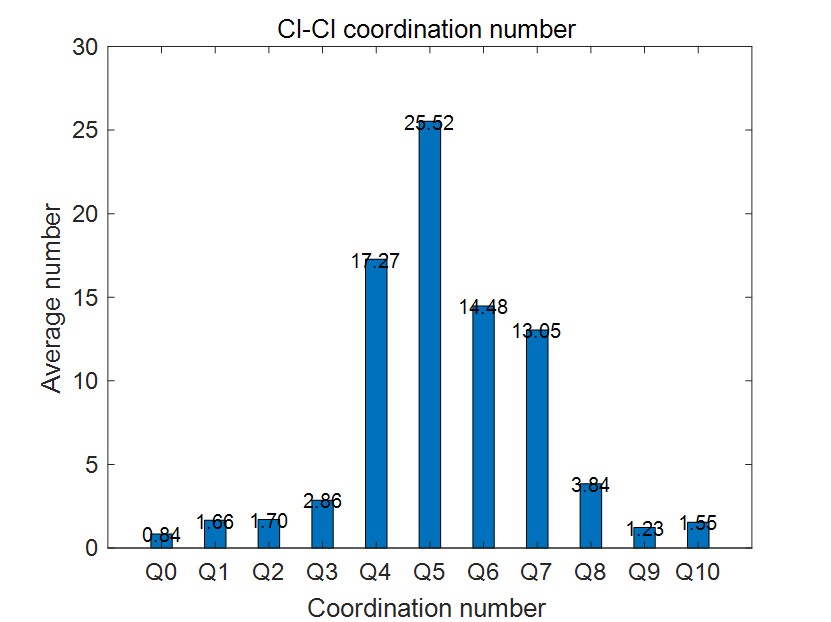

Supplement: Supplementary file 1 [file materials-19-01988-s001.zip › Figure S1/30molCaI2/973K/ClCl.jpg]

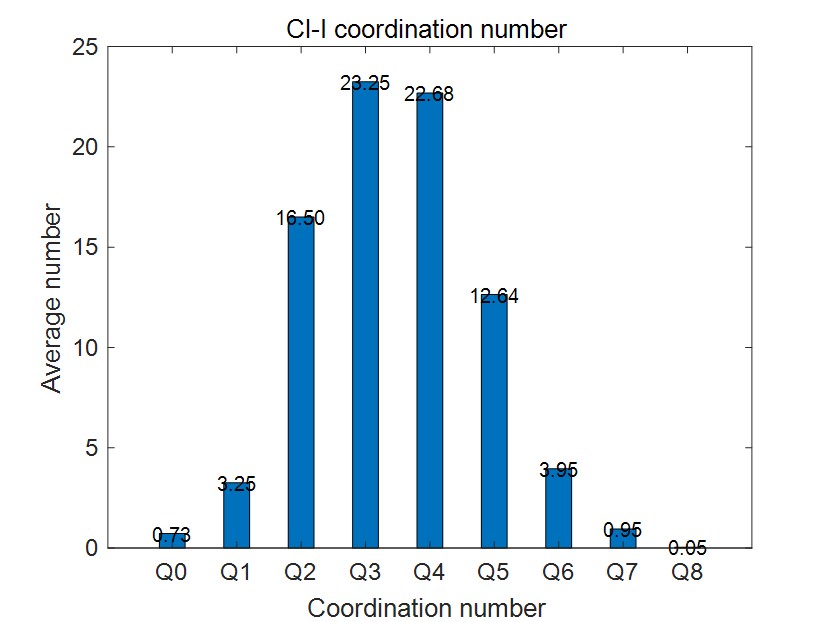

Supplement: Supplementary file 1 [file materials-19-01988-s001.zip › Figure S1/30molCaI2/973K/ClI.jpg]

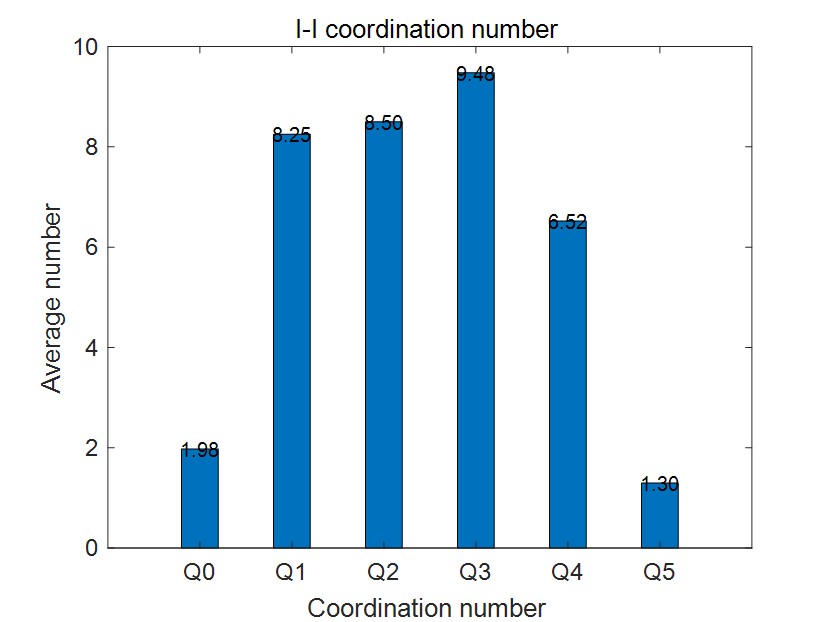

Supplement: Supplementary file 1 [file materials-19-01988-s001.zip › Figure S1/30molCaI2/973K/II.jpg]
